# CircRNA-CREIT inhibits stress granule assembly and overcomes doxorubicin resistance in TNBC by destabilizing PKR

**DOI:** 10.1186/s13045-022-01345-w

**Published:** 2022-08-29

**Authors:** Xiaolong Wang, Tong Chen, Chen Li, Wenhao Li, Xianyong Zhou, Yaming Li, Dan Luo, Ning Zhang, Bing Chen, Lijuan Wang, Wenjing Zhao, Shanji Fu, Qifeng Yang

**Affiliations:** 1 Department of Breast Surgery, General Surgery, Qilu Hospital of Shandong University, No. 107 Wenhua Xi Road, Jinan, 250012 Shandong China; 2 Pathology Tissue Bank, Qilu Hospital of Shandong University, Jinan, Shandong China; 3 Department of Clinical Laboratory, Qilu Hospital of Shandong University, Jinan, Shandong China; 4 Research Institute of Breast Cancer, Shandong University, Jinan, Shandong China

**Keywords:** CircRNA-CREIT, TNBC, Stress granules, Chemoresistance

## Abstract

**Background:**

Circular RNAs (circRNAs) represent a novel type of regulatory RNA characterized by high evolutionary conservation and stability. CircRNAs are expected to be potential diagnostic biomarkers and therapeutic targets for a variety of malignancies. However, the regulatory functions and underlying mechanisms of circRNAs in triple-negative breast cancer (TNBC) are largely unknown.

**Methods:**

By using RNA high-throughput sequencing technology, qRT-PCR and in situ hybridization assays, we screened dysregulated circRNAs in breast cancer and TNBC tissues. Then in vitro assays, animal models and patient-derived organoids (PDOs) were utilized to explore the roles of the candidate circRNA in TNBC. To investigate the underlying mechanisms, RNA pull-down, RNA immunoprecipitation (RIP), co immunoprecipitation (co-IP) and Western blotting assays were carried out.

**Results:**

In this study, we demonstrated that circRNA-CREIT was aberrantly downregulated in doxorubicin resistant triple-negative breast cancer (TNBC) cells and associated with a poor prognosis. The RNA binding protein DHX9 was responsible for the reduction in circRNA-CREIT by interacting with the flanking inverted repeat Alu (IRAlu) sequences and inhibiting back-splicing. By utilizing in vitro assays, animal models and patient-derived organoids, we revealed that circRNA-CREIT overexpression significantly enhanced the doxorubicin sensitivity of TNBC cells. Mechanistically, circRNA-CREIT acted as a scaffold to facilitate the interaction between PKR and the E3 ligase HACE1 and promoted proteasomal degradation of PKR protein via K48-linked polyubiquitylation. A reduced PKR/eIF2α signaling axis was identified as a critical downstream effector of circRNA-CREIT, which attenuated the assembly of stress granules (SGs) to activate the RACK1/MTK1 apoptosis signaling pathway. Further investigations revealed that a combination of the SG inhibitor ISRIB and doxorubicin synergistically inhibited TNBC tumor growth. Besides, circRNA-CREIT could be packaged into exosomes and disseminate doxorubicin sensitivity among TNBC cells.

**Conclusions:**

Our study demonstrated that targeting circRNA-CREIT and SGs could serve as promising therapeutic strategies against TNBC chemoresistance.

**Supplementary Information:**

The online version contains supplementary material available at 10.1186/s13045-022-01345-w.

## Background

Globally, breast cancer (BC) is the most prevalent type of malignancy and the leading cause of cancer death in female [1]. Triple-negative breast cancer (TNBC), which accounts for approximately 15–20% of all breast cancers, is characterized by a lack of estrogen receptor (ER), progesterone receptor (PR) and human epidermal growth factor receptor 2 (HER2) [2]. TNBC is a cluster of heterogeneous and aggressive diseases associated with higher risk of recurrence, metastasis and death than other BC subtypes [3]. Due to the absence of the receptors mentioned above, the patients are unable to benefit from traditional endocrine therapy and HER2-targeted therapy. So far, chemotherapy is still the main option for TNBC treatment in both neoadjuvant and adjuvant settings [4].

Most widely used chemotherapeutics, such as doxorubicin (DOX), exert their cytotoxic effects by inducing DNA strand breakage or RNA metabolism defects [5]. According to clinical trials, the TNBC subtype is more sensitive to chemotherapy and has higher pathologic complete response (pCR) rates than non-TNBC subtypes [6]. A real-world study revealed that chemotherapy significantly increased the overall survival rate (adjusted HR = 0.58, 95% CI = 0.46–0.73) and breast cancer-specific survival rate (adjusted HR = 0.65, 95% CI = 0.48–0.89) of TNBC patients during the 8.2-year median follow-up [7, 8]. However, a sustained response can only be observed in a small proportion of TNBC patients and chemoresistance eventually develops in most patients. The acquisition of drug resistance is a multifactorial and complex process driven by a variety of mechanisms, such as increased cellular damage repair or reduced cell apoptosis [9]. Once chemoresistance occurs, recurrent disease can develop rapidly, which has become a major obstacle in treating TNBC. Therefore, it is urgently necessary to identify novel therapeutic targets to overcome the chemoresistance in TNBC.

Over the last few years, the intimate relationship between stress granules (SGs) and chemoresistance has been revealed [10]. SGs are membraneless cytosolic compartments formed by liquid–liquid phase separation (LLPS) in response to multiple cellular stresses [11]. Phosphorylation of eukaryotic initiation factor 2 alpha (eIF2α) at serine residue 51 is a major trigger of SG formation by arresting translation initiation in response to stress. Then, the mRNAs released from the disassembled polysomes can be recruited to SGs for transient storage and remain translationally silent [12]. The Formation of SGs exerts essential roles in metabolic regulation to protect cells from harmful conditions. For instance, SGs can inhibit the activation of the stress-induced apoptosis pathway by sequestering the signaling protein RACK1 (Receptor for activated C kinase 1) and preventing the binding between RACK1 and MTK1 (mitogen-activated protein kinase kinase kinase 4) [13]. The roles of SGs in cancer progression particularly in chemoresistance have been increasingly concerned. Recent studies demonstrated that chemotherapeutic agents such as 5-Fu and capecitabine could induce drug resistance via excessive formation of SGs in cancer cells [10, 14]. Therefore, targeting SG formation might serve as an effective modality to enhance the efficacy of chemotherapy in cancer. However, the roles of SGs in regulating the chemosensitivity of TNBC cells have rarely been reported.

Circular RNA (circRNA) is an emerging class of endogenous RNA transcripts with a covalently closed loop structure. In contrast to the linear RNAs, circRNAs have no 5’ caps or 3’ tails and are characterized by a longer half-life, higher evolutionary conservation and more resistance to RNase R digestion [15]. CircRNAs can affect gene expression through a variety of mechanisms, such as functioning as miRNA sponges or interacting with proteins [16]. Accumulating evidence has highlighted the indispensable regulatory role of circRNAs in carcinogenesis and development. For instance, hsa_circ_0000190 was reported to be overexpressed in non-small cell lung carcinoma (NSCLC) and promote NSCLC progression via activation of the EGFR/ERK pathway [17]. We previously reported that circHIF1A could facilitate TNBC proliferation and metastasis by increasing the expression level of NFIB [18]. However, the functions and underlying mechanisms of circRNAs in regulating chemoresistance of TNBC are largely unknown. Therefore, identifying circRNAs that possess regulatory roles in chemosensitivity and investigating the underlying mechanisms might provide novel therapeutic targets for TNBC treatment.

In our present study, we found that circRNA-CREIT (a circRNA acting as a chemoresistance inhibitor in TNBC, circBase ID: hsa_circ_0001798) played an essential role in attenuating chemoresistance in TNBC. CircRNA-CREIT was significantly downregulated in chemoresistant breast cancer cells and ectopic overexpression of circRNA-CREIT substantially increased chemotherapy-induced apoptosis. Mechanistically, circRNA-CREIT promoted the degradation of PKR through HACE1-mediated ubiquitin–proteasome pathway, subsequently suppressing the phosphorylation of eIF2α and the assembly of SGs. Inhibited SG formation by circRNA-CREIT allowed more RACK1 protein to interact with MTK1 and activate the apoptosis pathway. The combination of chemotherapy and ISRIB, a small molecule that inhibits SG formation [19], exerted synergistic effects in sensitizing TNBC cells to chemotherapy. Moreover, circRNA-CREIT could be packaged into exosomes and exert its functions by exosome transmission. Our findings demonstrated that circRNA played an important role in modulating SG formation and that suppression of SGs significantly enhanced TNBC chemosensitivity.

## Methods and materials

### Patients and tissue samples

Two independent cohorts of 379 breast cancer patients in total were included in this study. Cohort 1 consisted of 321 breast cancer patients and this cohort was used for analysis of the relationship between circRNA-CREIT expression and the clinicopathological features of the patients. Among them, 244 patients with complete prognostic information were used for the survival analysis. The clinicopathological features of cohort 1 are shown in Additional file 1: Table S1. Cohort 2 comprised 58 breast cancer patients who received DOX-based chemotherapy after surgery. In cohort 2, twenty-one patients manifesting early relapse within 6 months after the last course of chemotherapy were defined as the chemotherapy-resistant group (subgroup 1) and thirty-seven patients with no recurrent disease during follow-up comprised the chemotherapy-sensitive group (subgroup 2), in accordance with previous studies [20]. The clinicopathological features of the two subgroups were matched, such as age, menstrual state, pathological stage and lymph node status, as shown in Additional file 1: Table S2. The tissue samples of cohort 2 were used to detect the expression of circRNA-CREIT in the two subgroups. All patients underwent surgery at the Department of Breast Surgery, Qilu Hospital, between 2008 and 2019. The tumor samples and adjacent normal counterparts were all collected during surgery. None of the patients were treated with chemotherapy or other related therapies in prior to surgery. Male patients and those with metastatic disease or other malignancies were excluded. All of the collected tissue samples were pathologically confirmed by three pathologists and stored at − 80 ℃ until use. Our study was approved by the Ethics Committee on Scientific Research of Shandong University, Qilu Hospital, and informed consent was obtained from all of the patients participating in the study.

### Cell culture and treatments

Human HEK-293T cells, the human breast cancer cell lines MDA-MB-231, MDA-MB-468, MDA-MB-436, MCF-7, ZR-75-1, SKBR3 and HS578T, and the human normal mammary epithelial cell line MCF10A were purchased from American Type Culture Collection (ATCC, VA, USA). These cell lines were regularly authenticated by STR analysis and checked for mycoplasma contamination. For the culture of MDA-MB-231, MDA-MB-468, HS578T and HEK-293T cells, high glucose DMEM (Macgene, Beijing, China) supplemented with 10% fetal bovine serum (Gemini, CA, USA), 100 U/ml penicillin (Macgene, Beijing, China) and 100 μg/ml streptomycin (Macgene, Beijing, China) were used. MDA-MB-436, MCF-7, ZR-75-1 and SKBR3 cells were maintained in RPMI-1640 (Macgene, Beijing, China) supplemented with 10% fetal bovine serum (Gemini, CA, USA), 100 U/ml penicillin (Macgene, Beijing, China) and 100 μg/ml streptomycin (Macgene, Beijing, China). MCF-10A cells were cultured as previously described [21]. All of the cells were incubated at 37 ℃ in a humidified atmosphere with 5% CO2. To inhibit RNA or protein synthesis, cells were treated with actinomycin D (Act D, CST, MA, USA) or cycloheximide (CHX, Selleck, TX, USA), respectively, for the indicated periods of time. MG132 (Selleck, TX, USA) was used to inhibit protein degradation via the proteasome pathway.

### In situ hybridization (ISH) assay

A specific digoxin-labeled circRNA-CREIT probe was designed, and the targeted sequence of the probe is shown in Additional file 1: Table S3. The tumor samples were fixed with formalin, embedded in paraffin and then sectioned into 6-µm slides. The ISH assay was conducted with the Enhanced Sensitive ISH Detection kit I (BOSTER, Wuhan, China) according to the manufacturer’s protocol. The deparaffinization and rehydration of the sections were the same as for the process of IHC assay.

### Statistical analysis

GraphPad Prism V8.3.0 was used for statistical analyses and the data were presented as the mean ± standard deviation (S.D.). Unpaired two-tailed Student’s t-test was used to compare the differences of cell viability, cell migration and invasion ability, cell apoptosis level, xenograft tumor volume, xenograft tumor weight and gene expression between the different groups. Paired two-tailed Student’s t test was used to compare the expression of circRNA-CREIT between breast cancer tissues and paired adjacent normal mammary tissues. The chi-square test was used to analyze the relationship between circRNA-CREIT expression and the clinicopathological features of the patients. Kaplan–Meier curves and log-rank tests were applied to compare the survival of patients with high and low circRNA-CREIT expression. In our study, *p* < 0.05 was considered statistically significant.

Detailed methods for the in vivo experiments and the other in vitro experiments are described in Additional file 1 .

## Results

### circRNA-CREIT was downregulated in TNBC

**Fig. 1:**
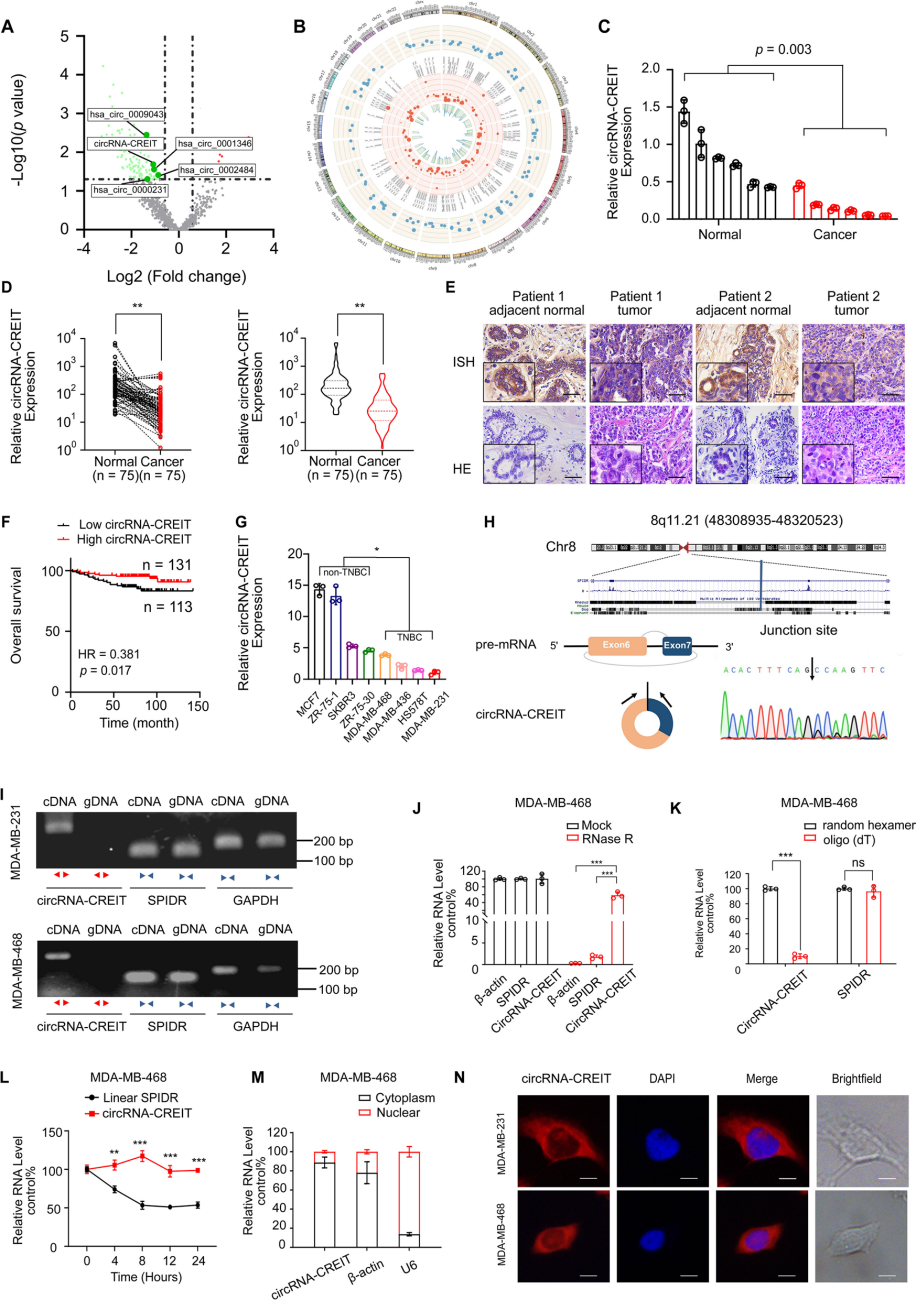
CircRNA-CREIT was downregulated in TNBC. **A** A volcano plot showing the upregulated and downregulated circRNAs in breast cancer tissues. **B** Circos plot indicating the differentially expressed circRNAs. The outermost circle shows the chromosomal distribution of the circRNAs. The second circle shows the expression levels of the indicated circRNAs. The third circle indicates the circBase ID of the circRNAs. The fourth circle shows the logFC of the indicated circRNAs between the two groups. The innermost circle shows the log*p* values. **C** qRT-PCR assay was performed to detect the expression of circRNA-CREIT in the 6 paired breast cancer tissues and their normal counterparts. **D** Expression of circRNA-CREIT in 75 breast cancer tissues and normal mammary tissues detected by qRT-PCR assay. **E** ISH of circRNA-CREIT in breast cancer tissues and paired normal mammary tissues. Scale bars = 200 μm. **F** Kaplan–Meier survival analysis of circRNA-CREIT^high^ and circRNA-CREIT^low^ patients. **G** Expression of circRNA-CREIT in breast cancer cell lines with different hormone receptor statuses. **H** (upper) Diagram showing the locus of circRNA-CREIT in the genome. (lower) Schematic illustration showing that exons 6–7 of human SPIDR circularize to form circRNA-CREIT. The black arrow represents the back-splicing site of circRNA-CREIT confirmed by Sanger sequencing. **I** Convergent and divergent primers were used to validate the loop structure of circRNA-CREIT. **J** The expression of circRNA-CREIT, linear SPIDR and β-actin in TNBC cells with or without RNase R treatment. **K** qRT-PCR analysis of circRNA-CREIT expression in cDNA reverse transcribed with random hexamer or oligo (dT) primers. **L** Relative RNA levels of circRNA-CREIT and linear SPIDR after actinomycin D treatment detected by qRT-PCR. **M** Detection of circRNA-CREIT expression in cytoplasmic and nuclear fractions of RNAs extracted from TNBC cells. **N** RNA FISH assay for circRNA-CREIT with the nucleus distinguished by DAPI. Scale bars = 10 μm. *ns* no significance; *p < 0.05; **p < 0.01; ***p < 0.001 compared with the controls

To identify the dysregulated circRNAs in human breast cancer, we profiled circRNA transcripts from six paired breast cancer tissues and adjacent normal mammary tissues using RNA-sequencing (RNA-seq) analysis. As shown in Fig. 1A, B, there were 108 differentially expressed circRNAs between the two groups with the criteria of fold change > 1.5 and *p* value < 0.05. Then, five dysregulated circRNAs (hsa_circ_0001346, hsa_circ_0002484, hsa_circ_0000231, hsa_circ_0009043 and circRNA-CREIT (circBase ID: hsa_circ_0001798)) with high abundance were selected for further validation. Among them, the expression of circRNA-CREIT showed the most remarkable difference between the breast cancer tissues and paired normal counterparts (Fig. 1C, D and Additional file 1: Fig. S1A). The ISH assay also verified the significant downregulation of circRNA-CREIT in breast cancer tissues (Fig. 1E).

Next, 244 breast cancer patients were divided into high circRNA-CREIT expression group (*n* = 131) and low circRNA-CREIT expression group (*n* = 113). Kaplan–Meier plotter demonstrated that high expression of circRNA-CREIT was associated with a favorable prognosis (Fig. 1F). Multivariate analyses revealed that circRNA-CREIT was an independent predictive factor for the prognosis of breast cancer patients (Additional file 1: Table S4). Moreover, higher levels of circRNA-CREIT were significantly correlated with a lower pathological grade, a lower rate of lymph node metastasis and a smaller tumor size (Additional file 1: Fig. S1B-D). We then detected the expression of circRNA-CREIT in breast cancer cell lines and found that circRNA-CREIT was much lower in TNBC than in the non-TNBC subtype (Fig. 1G). Collectively, we speculated that circRNA-CREIT might play a suppressive role in the progression of breast cancer, especially in the TNBC subtype.

### Characteristics of circRNA-CREIT in TNBC

CircRNA-CREIT is derived from the exons 6–7 of the *SPIDR* (scaffold protein involved in DNA repair) gene located on human chromosome 8. The back-splicing site was confirmed by Sanger sequencing in TNBC cells (Fig. 1H). To validate the circular structure of circRNA-CREIT, we designed convergent and divergent primers to amplify circRNA-CREIT and linear SPIDR, respectively. As shown in Fig. 1I, circRNA-CREIT could only be amplified from cDNA rather than gDNA, indicating that circRNA-CREIT was a back-splicing product of the pre-mRNA. Additionally, circRNA-CREIT was more resistant to RNase R degradation than the linear form of SPIDR mRNA (Fig. 1J, Additional file 1: Fig. S1E). Moreover, circRNA-CREIT could not be efficiently reverse-transcribed with oligo (dT) primers, consistent with its loop structure lacking a poly-A tail (Fig. 1K and Additional file 1: Fig. S1F). The actinomycin D assay demonstrated that the half-life of circRNA-CREIT was much longer than that of the linear SPIDR transcript, which verified the high stability of circRNA-CREIT (Fig. 1L and Additional file 1: Fig. S1G). Additionally, qRT‒PCR and fluorescence in situ hybridization (FISH) assays showed the predominant cytoplasmic distribution of circRNA-CREIT (Fig. 1M, N and Additional file 1: Fig. S1H).

The expressions of circRNAs usually depend on the expression levels of their host genes and back-splicing rate [22]. To explore the mechanisms underlying circRNA-CREIT downregulation, we detected the SPIDR levels in tumor tissues. As shown in Additional file 1: Fig. S2A, B, SPIDR was significantly increased in breast cancer tissues compared to their normal counterparts, which was not responsible for the downregulation of circRNA-CREIT. Therefore, we determined to investigate the roles of back-splicing in circRNA-CREIT biogenesis. It was previously reported that DExH-box helicase 9 (DHX9), a well-known RNA helicase, could bind with the inverted repeat Alu (IRAlu) sequence located in the flanking introns to unwind IRAlu pairs and reduce the biogenesis of circRNAs [23]. Our results demonstrated that DHX9 overexpression led to the downregulation of circRNA-CREIT, while silencing DHX9 increased its levels (Additional file 1: Fig. S2C). In addition, by analyzing the data from the TCGA and GEO databases, we found that DHX9 was significantly upregulated in breast cancer, especially in the TNBC subtype, and was related to the poor overall survival of patients (Additional file 1: Fig. S2D-G). Then the sequences of the flanking introns were compared to the Alu sequence using the BLAST tools. We found twenty Alu elements in intron 5 and five Alu elements in intron 7. Previous studies revealed that the Alu element targeted by DHX9 had a much shorter distance to its closest potential pairing partner than that not targeted [24]. Therefore, we selected the closest complementary IRAlu pair (Alu19 and Alu 21) for further investigation (Additional file 1: Fig. S2H). RIP assays confirmed the interaction of the DHX9 protein and the two Alu elements (Additional file 1: Fig. S2I).

### CircRNA-CREIT enhanced DOX sensitivity of TNBC in vitro and in vivo

**Fig. 2:**
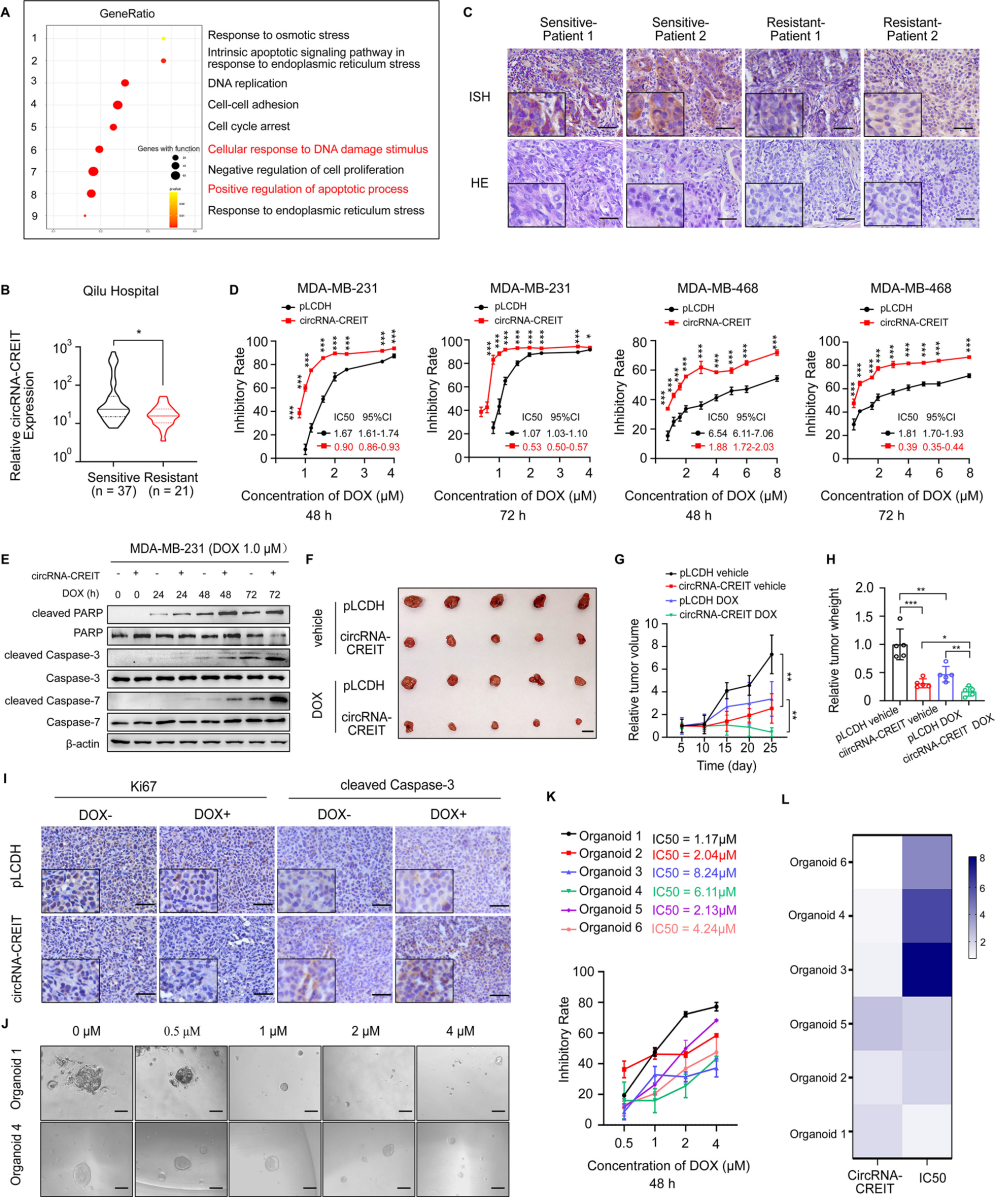
CircRNA-CREIT significantly enhanced the chemosensitivity of TNBC cells in vitro and in vivo. **A** GO analysis of differentially expressed genes in circRNA-CREIT-overexpressing MDA-MB-231 cells based on RNA-seq data. **B** Violin plot showing circRNA-CREIT expression in chemosensitive and chemoresistant breast cancer tissues, detected by qRT-PCR assays. **C** ISH assay of circRNA-CREIT in chemosensitive and chemoresistant breast cancer tissues. Scale bars = 200 μm. **D** The impact of circRNA-CREIT overexpression on the cytotoxic effects of DOX is shown. Cell viability was detected by MTT assays. The IC50 values with the 95% CIs are presented. **E** Western blotting was performed to detect the expression of apoptosis pathway markers. **F** Images of the xenograft tumors. Scale bars = 10 mm. **G** Growth curves of xenograft tumors in different groups after treatment. **H** The tumor weights of the xenograft tumors. **I** Representative images of IHC staining for Ki67 and cleaved caspase-3 in different groups. Scale bars = 100 μm. **J** Representative morphologies of breast cancer patient-derived organoids (PDOs) treated with increasing DOX concentrations. Scale bars = 200 μm. **K** The IC50 values and 95% CIs of the PDOs are shown. Cell viability was measured by CCK8 assays. **L** Heatmap showing the IC50 value and relative circRNA-CREIT expression level of each PDO. Three independent experiments were conducted for each result. *p < 0.05; **p < 0.01; ***p < 0.001 compared with the controls

To explore the potential roles of circRNA-CREIT in TNBC, we constructed circRNA-CREIT-overexpressing plasmids and three different short hairpin RNAs (shRNAs) targeting the junction region. The overexpression and knockdown efficiencies were validated in TNBC cells (Additional file 1: Fig. S3A, B). We found that shRNA-3 had a better knockdown effect, and it was used in our subsequent studies. Moreover, the expression of host gene SPIDR was not influenced by the change of circRNA-CREIT levels (Additional file 1: Fig. S3C, D). Then we applied RNA-seq to screen differentially expressed genes in circRNA-CREIT overexpressing cells (Additional file 1: Fig. S4A). Gene Ontology (GO) analysis and Gene Set Enrichment Analysis (GSEA) for differentially expressed genes revealed that circRNA-CREIT might be closely associated with the regulation of cellular response to various environmental stresses, especially chemical-induced stresses and the process of apoptosis (Fig. 2A and Additional file 1: Fig. S4B). DOX is the first-line chemotherapeutic agent in TNBC treatment and has potent antitumor effects [25]. Thus, DOX was used in our following experiments. By conducting qRT-PCR and ISH assays, we found that circRNA-CREIT was significantly downregulated in chemotherapy-resistant breast cancer tissues and doxorubicin resistant TNBC cells (MDA-MB-231/DOXR) (Fig. 2B, C and Additional file 1: Fig. S4C, D). As shown in Fig. 2D and Additional file 1: Fig. S5A, circRNA-CREIT overexpression obviously improved the chemosensitivity of TNBC cells to DOX, with a significant decrease in the IC50 value, whereas reduced circRNA-CREIT expression levels had the opposite effect. The results of the colony formation assay were consistent with the above findings (Additional file 1: Fig. S5B). In addition, we found that circRNA-CREIT overexpression notably enhanced DOX-induced apoptosis, while circRNA-CREIT knockdown led to decreased cell apoptosis (Fig. 2E and Additional file 1: Fig. S5C, D).

To evaluate the effect of circRNA-CREIT on tumor chemosensitivity in vivo, tumor xenograft models were constructed in female nude BALB/c mice. The results showed that the tumor growth rate and tumor weight were significantly decreased by circRNA-CREIT overexpression in both the vehicle control group and the DOX-treated group (Fig. 2F–H). Immunohistochemistry analysis for Ki67 and cleaved caspase-3 was performed to detect the proliferative rate and apoptosis level in the xenograft tumors. Decreased proliferation and elevated apoptosis levels were observed in the circRNA-CREIT overexpression group, especially in the DOX-treated group (Fig. 2I). Conversely, circRNA-CREIT knockdown led to accelerated tumor growth, reduced chemosensitivity of tumors to DOX and downregulated apoptosis (Additional file 1: Fig. S5E-H). We then constructed a patient-derived organoid (PDO) model to detect the correlation between circRNA-CREIT expression and PDO sensitivity to doxorubicin. The results showed that PDOs with higher expression levels of circRNA-CREIT tended to be more sensitive to DOX and had a lower IC50 values (Fig. 2J–L and Additional file 1: Fig. S5I).

GO and GSEA analyses (Fig. 2A, and Additional file 1: Fig. S6A) also suggested circRNA-CREIT might play a role in cell proliferation, cell adhesion and epithelial–mesenchymal transition (EMT), and hence, we tested the function of circRNA-CREIT in the above biological processes. The results demonstrated that circRNA-CREIT overexpression could significantly inhibit cell proliferation, migration, invasion and EMT in TNBC cells, in contrast to the effect of circRNA-CREIT knockdown (Additional file 1: Fig. S6B-L).

### CircRNA-CREIT directly interacted with double-stranded RNA-activated protein kinase (PKR)

**Fig. 3:**
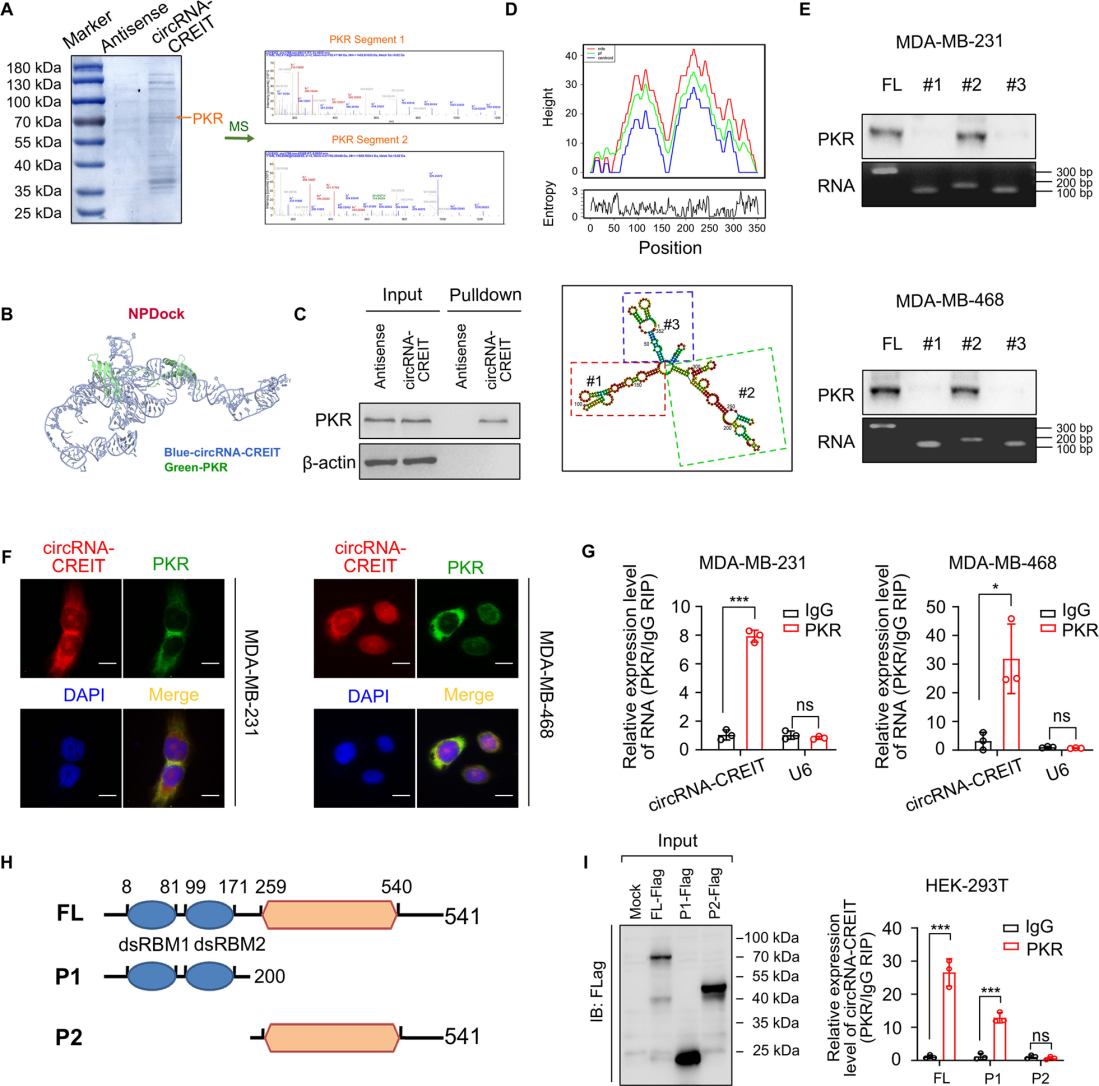
CircRNA-CREIT physically interacted with PKR. **A** CircRNA-CREIT probes and control probes were biotinylated and incubated with MDA-MB-231 cell lysates for RNA pull-down assays. (left) Photograph presenting Coomassie brilliant blue staining for the proteins precipitated in the RNA pull-down assays. The orange arrow indicates the size of the PKR protein. (right) Two segments of PKR proteins identified by mass spectrometry (MS). **B** Graphical representation of the molecular docking between circRNA-CREIT and the PKR protein using NPDock. **C** Western blotting of independent RNA pull-down assays verified the specific association of PKR protein with circRNA-CREIT using MDA-MB-231 cells. **D** The secondary structure of circRNA-CREIT was predicted by the online tool RNAfold web server (lower). (upper) Mountain plot representing the minimum free energy (MFE, red), the thermodynamic ensemble (green) and the centroid structures (blue) of circRNA-CREIT. CircRNA-CREIT was divided into three truncates representing three stem loop structures. **E** Western blotting analysis of PKR pulled down by different circRNA-CREIT truncates. **F** FISH and IF assays showing the colocalization of PKR and circRNA-CREIT. Scale bars = 10 μm. **G** RIP assay verifying the binding between PKR and circRNA-CREIT. **H** Diagrams of full-length (FL) PKR proteins and truncates with domain depletion. **I** (left) Western blotting analysis of PKR with full-length and truncated PKR proteins in the lysates of the HEK-293 T cells. (right) RIP assay for circRNA-CREIT enrichment in cells transfected with flag-tagged PKR (FL) overexpression vectors and truncated PKR expression vectors. Three independent experiments were conducted for each result. ns, no significance; *p < 0.05; ***p < 0.001 compared with the controls

To elucidate the molecular mechanisms by which circRNA-CREIT influences the chemosensitivity, we performed RNA pulldown assays, followed by electrophoresis, Coomassie blue staining and mass spectrometry (MS). Among the proteins identified by MS, PKR, which plays important roles in sensing and responding to multiple types of cellular stresses and cancer progression [26], aroused our interest and was identified as a candidate protein interacting with circRNA-CREIT (Fig. 3A). Then NPDock was used to predict the molecular docking between circRNA-CREIT and PKR protein, indicating the physical interaction between the two molecules (Fig. 3B). The binding between circRNA-CREIT and PKR was verified by independent RNA pulldown and immunoblot assays (Fig. 3C). To determine the specific region of circRNA-CREIT that mediated the association with PKR, the secondary structure of circRNA-CREIT with the minimum free energy (MFE) was predicted utilizing the online tool RNAfold (Fig. 3D, upper). Three truncated circRNA-CREIT probes representing three different stem-loops were constructed (Fig. 3D, lower). As shown in Fig. 3E, stem-loop #2 could efficiently pull down endogenous PKR protein in TNBC cells, whereas stem-loops #1 and #3 scarcely bound with PKR. FISH combined with immunofluorescent (IF) assays showed the colocalization of endogenous circRNA-CREIT and PKR in the cytoplasm, which further verified the direct interaction between circRNA-CREIT and PKR (Fig. 3F). Furthermore, we performed RNA immunoprecipitation (RIP) assays and observed marked enrichment of circRNA-CREIT using anti-PKR antibodies (Fig. 3G). To determine which domain of PKR was responsible for its association with circRNA-CREIT, we constructed truncated PKR mutants (Fig. 3H). RIP assays showed that deletion of the dsRNA-binding motif (dsRBM1-2) at the N-terminus significantly abolished the interaction between PKR and circRNA-CREIT (Fig. 3I).

### CircRNA-CREIT enhanced the degradation of PKR via K48-linked polyubiquitination

**Fig. 4:**
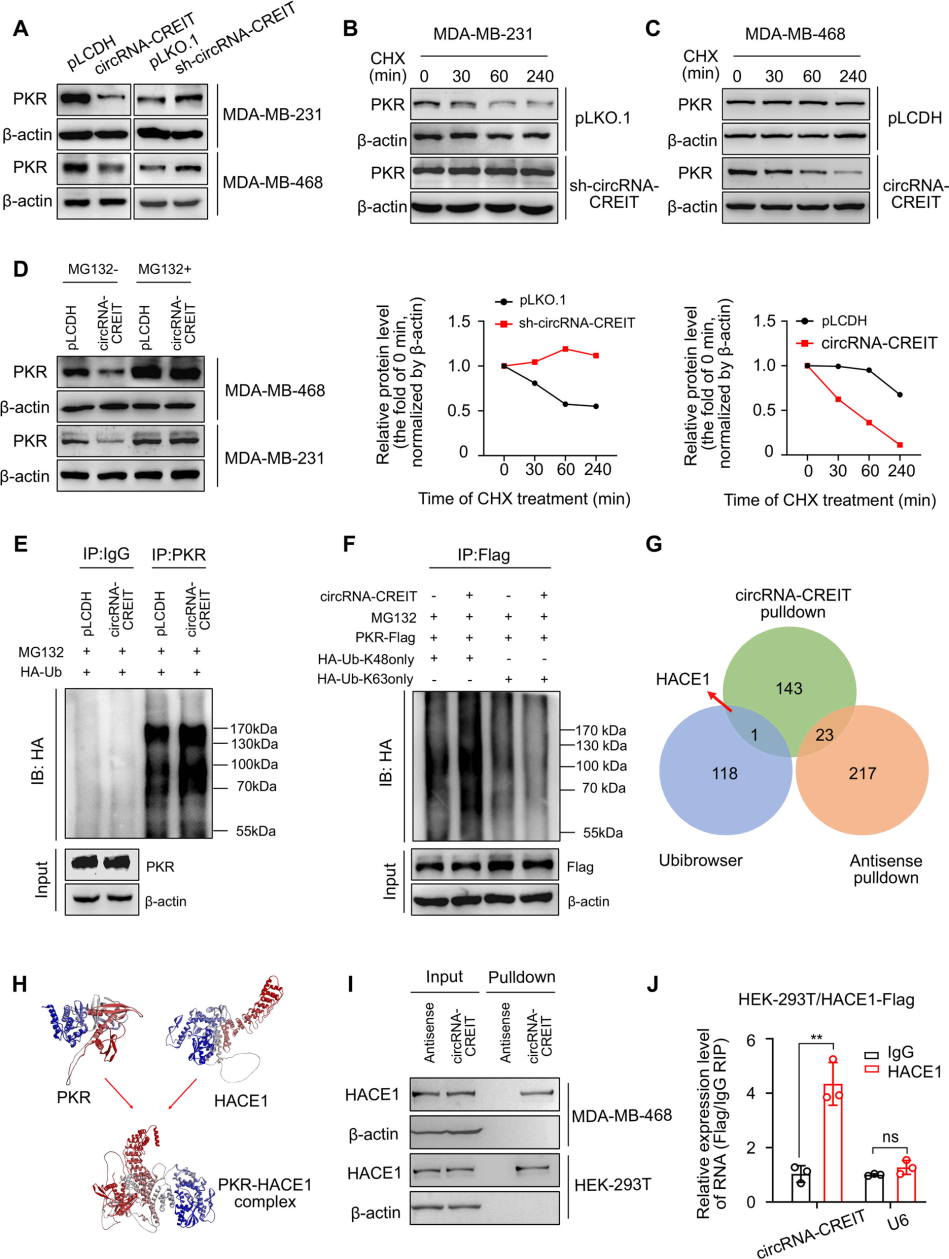
CircRNA-CREIT promoted PKR degradation via the ubiquitin–proteasome system. **A** Western blotting for PKR protein levels after circRNA-CREIT overexpression or knockdown. **B**, **C** TNBC cells with circRNA-CREIT overexpression or knockdown were treated with cycloheximide (CHX) for the indicated times. Western blotting analysis (upper) and statistical analysis (lower) of PKR levels upon CHX treatment are presented, with the level at 0 h as a control. **D** Western blotting analysis of PKR protein levels regulated by circRNA-CREIT with or without MG132 treatment. **E** Effects of circRNA-CREIT overexpression on the ubiquitination of PKR proteins. MDA-MB-231 cells were cotransfected with circRNA-CREIT overexpression plasmids and HA-tagged ubiquitin expression plasmids or the corresponding empty vectors. The cell lysates were incubated with anti-PKR or anti-IgG antibodies and protein A/G magnetic beads. The proteins precipitated in the co-IP assay were analyzed by Western blotting. *IB* immunoblot. **F** Western blotting assay showing the upregulation of K48-linked ubiquitination in circRNA-CREIT-overexpressing MDA-MB-231 cells. IB: immunoblot. HA-Ub-K48only: the cells were transfected with plasmids expressing HA-tagged ubiquitin with all lysines mutated except K48. HA-Ub-K63only: the cells were transfected with plasmids expressing HA-tagged ubiquitin with all lysines mutated except K63. **G** Identification of the candidate E3 ligases of PKR by bioinformatics prediction and MS analysis for RNA pull-down products, illustrated by a Venn diagram. **H** The interaction between PKR and HACE1 proteins was predicted by the ZDOCK server. The predicted structure was visualized by Discovery Studio software. **I**, **J** The interaction between circRNA-CREIT and HACE1 was verified by RNA pull-down assays (**I**) and RIP assay (**J**). Three independent experiments were conducted for each result. *ns* no significance; **p < 0.01 compared with the controls

We next investigated the impact of circRNA-CREIT on PKR expression. The results showed that the mRNA levels of PKR were not changed by circRNA-CREIT overexpression or knockdown (Additional file 1: Fig. S7A). However, elevated circRNA-CREIT expression led to significant downregulation of PKR protein, while circRNA-CREIT knockdown enhanced PKR expression (Fig. 4A). Further cycloheximide (CHX) treatment revealed that circRNA-CREIT knockdown increased the half-life of PKR protein, in contrast to the effect of circRNA-CREIT overexpression (Fig. 4B, C). Therefore, we hypothesized that circRNA-CREIT played an important role in PKR degradation.

In mammalian cells, the ubiquitin–proteasome system (UPS) participates in the degradation of most proteins [27]. To determine whether the UPS mediated the influence of circRNA-CREIT on PKR degradation, we treated cells with the proteasome inhibitor MG132. We found that MG132 abolished the effect of circRNA-CREIT overexpression on PKR protein levels (Fig. 4D). Then, co-IP assays were performed to detect the ubiquitination level of PKR proteins. We observed that the ubiquitination of endogenous PKR was markedly upregulated by circRNA-CREIT overexpression (Fig. 4E). K48- and K63-linked polyubiquitin chains are two main types of polyubiquitin linkage in mammalian cells [28]. K48-linked polyubiquitin usually induces degradation of substrate proteins and K63-linked polyubiquitin is correlated with protein stabilization or activation [29]. We observed that the K48-linked rather than K63-linked polyubiquitin of PKR was enhanced by circRNA-CREIT overexpression (Fig. 4F). These findings validated that circRNA-CREIT mediated PKR degradation through the K48-linked ubiquitin–proteasome pathway.

We next sought to determine which E3 ligases were involved in the regulatory effect of circRNA-CREIT on PKR degradation. The MS results revealed that there were several E3 ligases in the precipitation pulled down by circRNA-CREIT probes. Then, we predicted the potential E3 ligase of PKR by combining bioinformatics analysis on the Ubibrowser platform and the above MS results. The HECT domain and ankyrin repeat containing E3 ubiquitin protein ligase 1 (HACE1) was selected as the candidate (Fig. 4G). By applying molecular docking with ZDOCK (version 3.0.2) software, HACE1 was predicted to bind with PKR protein and the result was visualized by Discovery Studio (version 4.5) (Fig. 4H). In addition, circRNA-CREIT was verified to bind with HACE1, as indicated by RNA pulldown and RIP assays (Fig. 4I, J).

### CircRNA-CREIT enhanced HACE1-PKR interaction by serving as a scaffold

**Fig. 5:**
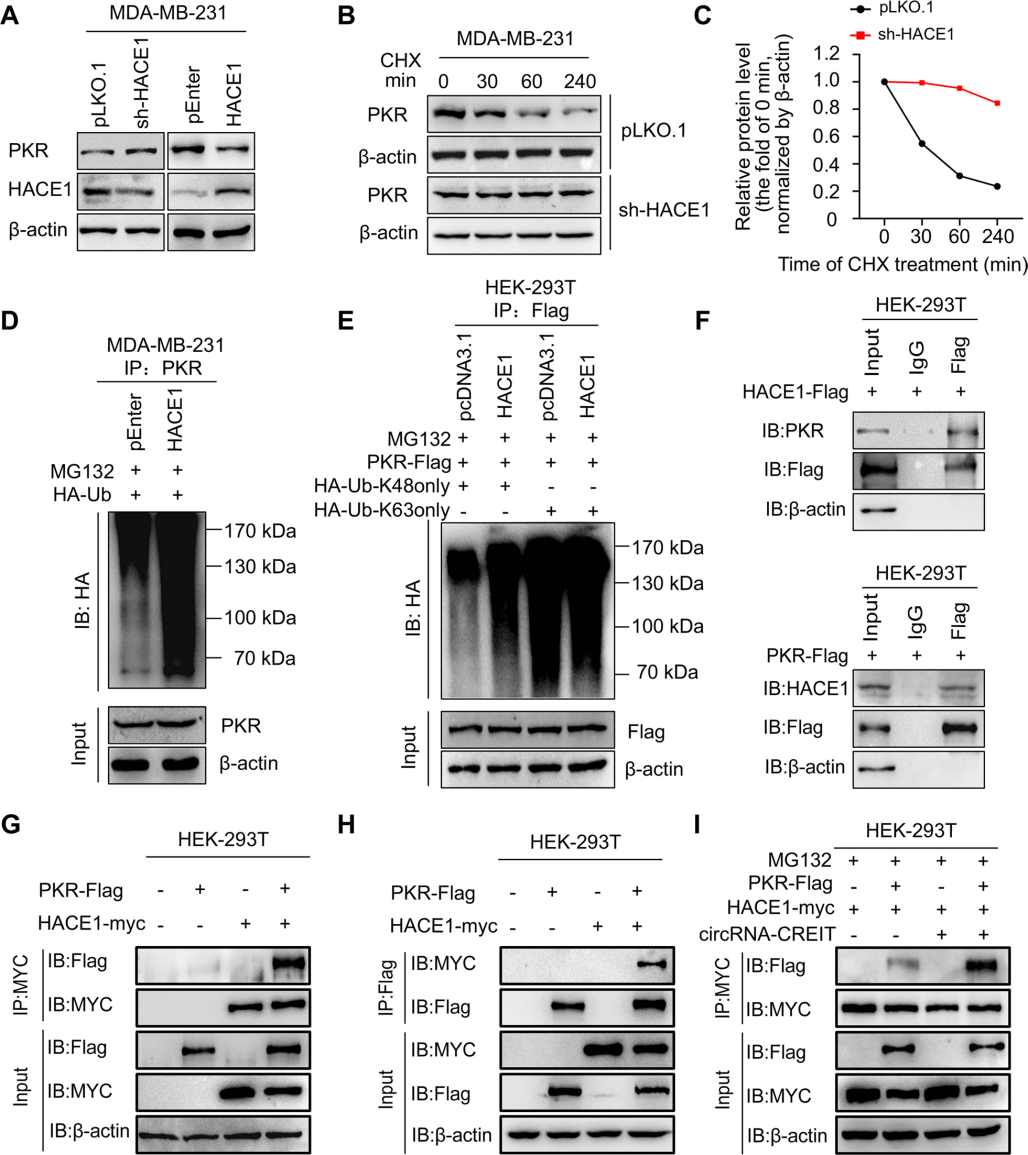
CircRNA-CREIT enhanced the binding of HACE1 and PKR proteins. **A** Western blotting for PKR protein levels after HACE1 overexpression or knockdown in TNBC cells. **B**, **C** TNBC cells stably transfected with sh-HACE1 plasmids or empty vectors were treated with CHX for the indicated times. Western blotting (**B**) and statistical analysis (**C**) of PKR protein levels are shown, with the level at 0 h as a control. **D** The impact of HACE1 on the PKR ubiquitination level was verified by co-IP assays and subsequent Western blotting. IB: immunoblot. **E** K48-linked ubiquitination of PKR was enhanced by HACE1 overexpression. IB: immunoblot. HA-Ub-K48only: the cells were transfected with plasmids expressing HA-tagged ubiquitin with all lysines mutated except K48. HA-Ub-K63only: the cells were transfected with plasmids expressing HA-tagged ubiquitin with all lysines mutated except K63. **F** HEK-293 T cells transfected with HACE1-Flag or PKR-Flag were lysed, immunoprecipitated with anti-Flag and then subjected to Western blotting assays using anti-PKR or anti-HACE1, respectively. **G**, **H** HEK-293 T cells co-transfected with PKR-Flag and HACE1-Myc or the corresponding empty vectors were lysed, immunoprecipitated with anti-MYC (**G**) or anti-Flag (**H**), and subjected to Western blotting analysis. **I** HEK-293 T cells were cotransfected with PKR-Flag, HACE1-Myc and circRNA-CREIT overexpression vectors or the corresponding empty plasmids. Co-IP assays validated that circRNA-CREIT increased the PKR-Flag level precipitated by HACE1-Myc. Three independent experiments were conducted for each result

Circular RNA can modulate the degradation process of downstream proteins by acting as a scaffold for that protein and its E3 ligase [30, 31]. According to the above results, we hypothesized that circRNA-CREIT might function in this way. As expected, HACE1 overexpression significantly inhibited PKR protein levels, and stable HACE1 knockdown increased PKR expression in TNBC cells (Fig. 5A). Additionally, HACE1 knockdown efficiently prolonged the half-life of PKR (Fig. 5B, C). Co-IP assays showed that cells with HACE1 overexpression exhibited increased PKR ubiquitylation, especially K48-linked ubiquitylation as expected (Fig. 5D, E). The above results provide essential evidence that HACE1 acts as an E3 ligase of PKR. Then, co-IP assays were performed to assess the interaction of PKR and HACE1. We observed that endogenous PKR could be immunoprecipitated by flag-tagged HACE1, and endogenous HACE1 could be immunoprecipitated by flag-tagged PKR (Fig. 5F). Moreover, exogenous co-IP assays demonstrated that PKR-flag could be easily detected in the anti-myc-HACE1 immunoprecipitates and vice versa (Fig. 5G, H). The interaction between PKR and HACE1 could be remarkably enhanced by circRNA-CREIT (Fig. 5I). Thus, circRNA-CREIT acted as a scaffold that could bring PKR and HACE1 together, leading to increased degradation of PKR.

### CircRNA-CREIT attenuated DOX-induced stress granule formation and released RACK1 trapped by stress granules via the PKR/eIF2α axis

**Fig. 6:**
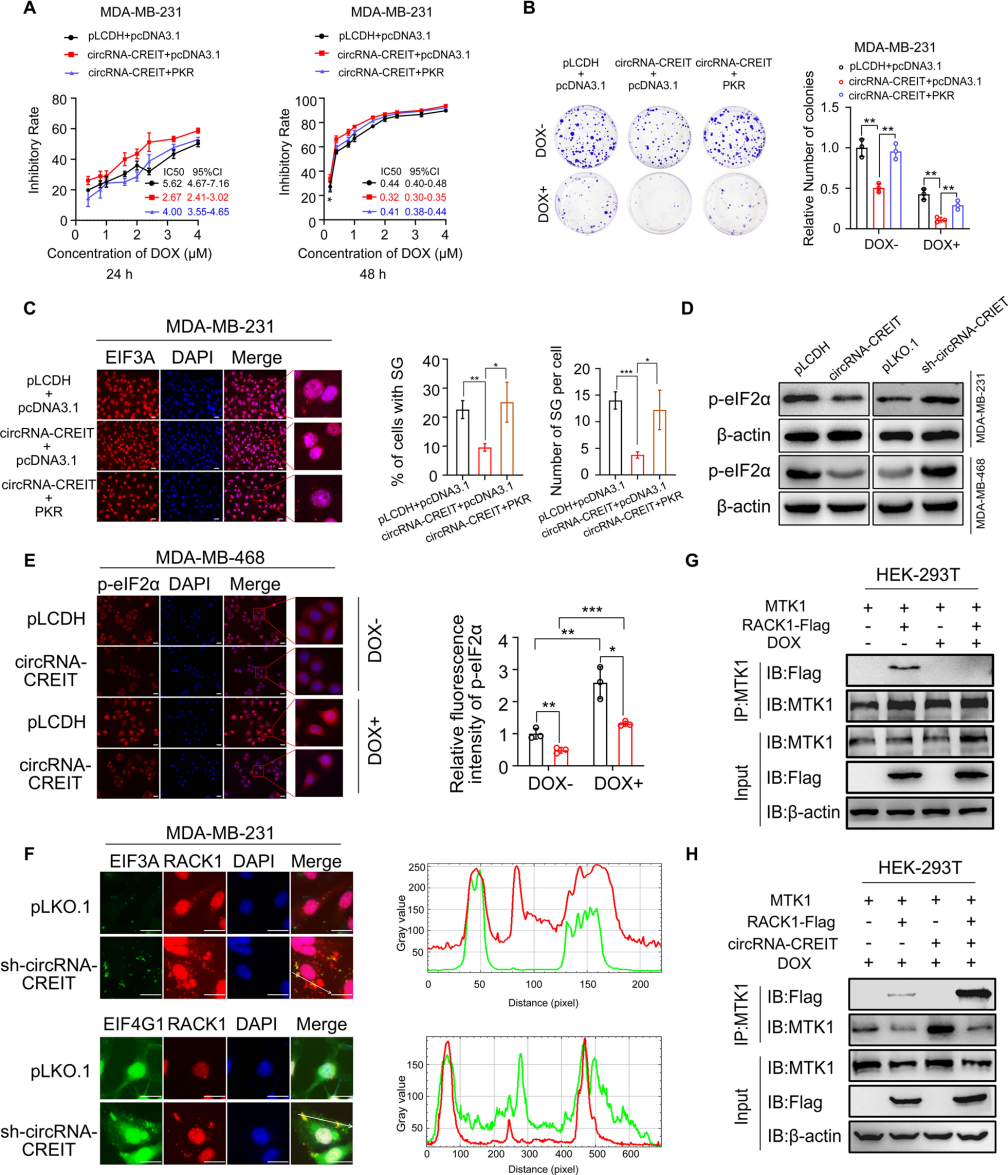
CircRNA-CREIT attenuated SG formation via the PKR/eIF2α axis. **A** PKR reversed the effects of circRNA-CREIT in promoting chemosensitivity. Cell viability was detected MTT assays. The IC50 and the 95% CI of cells with different treatments are shown. **B** Colony formation assays presenting the roles of PKR in mediating the functions of circRNA-CREIT. **C** Immunofluorescence staining for the SG marker EIF3A under DOX treatment (for 24 h). Representative images are shown and the percentage of cells with SGs and the number of SGs per cell were calculated. **D** Western blotting assay showing that circRNA-CREIT inhibited p-eIF2α expression and circRNA-CREIT knockdown increased p-eIF2α levels in TNBC cells. **E** Immunofluorescence staining for p-eIF2α after circRNA-CREIT overexpression or knockdown with or without DOX treatment (for 24 h). Quantitative analyses were performed using Image J, and the groups treated with empty vectors and PBS were used as controls. **F** Subcellular localization of RACK1 and endogenous SG markers EIF3A and EIF4G1 under DOX treatment. Cells were transfected with RACK1-pmCherry-C1 plasmids, treated with DOX for 24 h and subjected to immunofluorescence. Colocalization analysis for RACK1 and SG markers along the indicated line was performed by ImageJ. **G** Western blotting analysis of the co-IP assay showing that the interaction between RACK1 and MTK1 was blocked by DOX treatment. **H** Co-IP assay and the subsequent Western blotting assay verified that circRNA-CREIT restored the binding of RACK1 and MTK1. Scale bars = 20 μm. Three independent experiments were conducted for each result. *p < 0.05; **p < 0.01, ***p < 0.001 compared with the controls

According to previous reports, PKR is an important kinase that phosphorylates eIF2α at serine residue 51 and triggers the formation of stress granules (SGs) [32]. We examined whether circRNA-CREIT played its roles via the modulation of PKR expression. We found that TNBC cells overexpressing circRNA-CREIT exhibited significantly lower IC50 values of DOX than control cells, and this effect could be abrogated by PKR overexpression (Fig. 6A). In addition, we noticed that circRNA-CREIT knockdown and PKR overexpression played a synergistic role in elevating DOX resistance in TNBC cells (Additional file 1: Fig. S8A). Colony formation assays also confirmed the above findings (Fig. 6B and Additional file 1: Fig. S8B), and we concluded that PKR mediated the effect of circRNA-CREIT on chemosensitivity in TNBC cells.

Considering the notable roles of SGs in cancer progression, especially in enhancing drug resistance of cancer cells [32], we hypothesized that circRNA-CREIT might exert its effect on TNBC chemosensitivity by regulating the formation of SGs. By visualizing the SG marker proteins EIF3A, G3BP1 and G3BP2, we found that DOX treatment could efficiently trigger the formation of SGs (Additional file 1: Fig. S8C). As expected, circRNA-CREIT overexpression attenuated SG formation under DOX treatment, and this effect was rescued by PKR overexpression (Fig. 6C). In contrast, circRNA-CREIT knockdown remarkably enhanced the formation of DOX-induced and hypoxia-induced SGs (Additional file 1: Fig. S9A, B). Next, we examined whether circRNA-CREIT had an effect on the phosphorylation level of eIF2α. The results showed that circRNA-CREIT overexpression significantly reduced the phosphorylation of eIF2α, whereas circRNA-CREIT knockdown promoted eIF2α phosphorylation in TNBC cells (Fig. 6D, E and Additional file 1: Fig. S9C, D). DOX treatment triggered the phosphorylation of eIF2α in a time-dependent manner, and this effect could be enhanced by circRNA-CREIT knockdown (Additional file 1: Fig. S9E). Furthermore, we found that DOX could induce higher expression levels of p-eIF2α and more stress granules in DMDA-MB-231/DOXR than in MDA-MB-231 cells (Additional file 1: Fig. S10A, B). Therefore, there was a close relationship between DOX resistance and the SG formation in TNBC cells.

RACK1, a scaffold protein with multiple functions, plays an important role in activating stress-induced apoptosis by interacting with MTK1 in the cytoplasm. However, under specific cellular stresses, such as arsenite treatment, RACK1 can be sequestered into SGs and the interaction between RACK1 and MTK1 is blocked [13]. We then examined the influence of circRNA-CREIT on the subcellular locations of RACK1 under DOX treatment. We observed that in unstimulated TNBC cells, RACK1 was mainly diffusely distributed in the cytoplasm (data not shown). After treatment with DOX, most RACK1 proteins were transferred to the nucleus, while some condensed into granules in the cytoplasm. Intriguingly, circRNA-CREIT knockdown remarkably enhanced the nucleation of RACK1 proteins in the cytoplasm, many of which showed colocalization with SG markers (Fig. 6F and Additional file 1: Fig. S10C). These results suggested that circRNA-CREIT knockdown significantly promoted the recruitment of RACK1 by DOX-induced SGs. As shown in Fig. 6G, the binding of RACK1 and MTK1 was scarcely detected after DOX treatment, and was dramatically rescued by circRNA-CREIT overexpression (Fig. 6H). These results indicated that circRNA-CREIT could enhance stress-induced apoptosis and improve the chemosensitivity of TNBC cells by preventing RACK1 from being sequestered by SGs.

### The small molecule ISRIB targeting SGs greatly enhanced DOX sensitivity in synergy with circRNA-CREIT

**Fig. 7:**
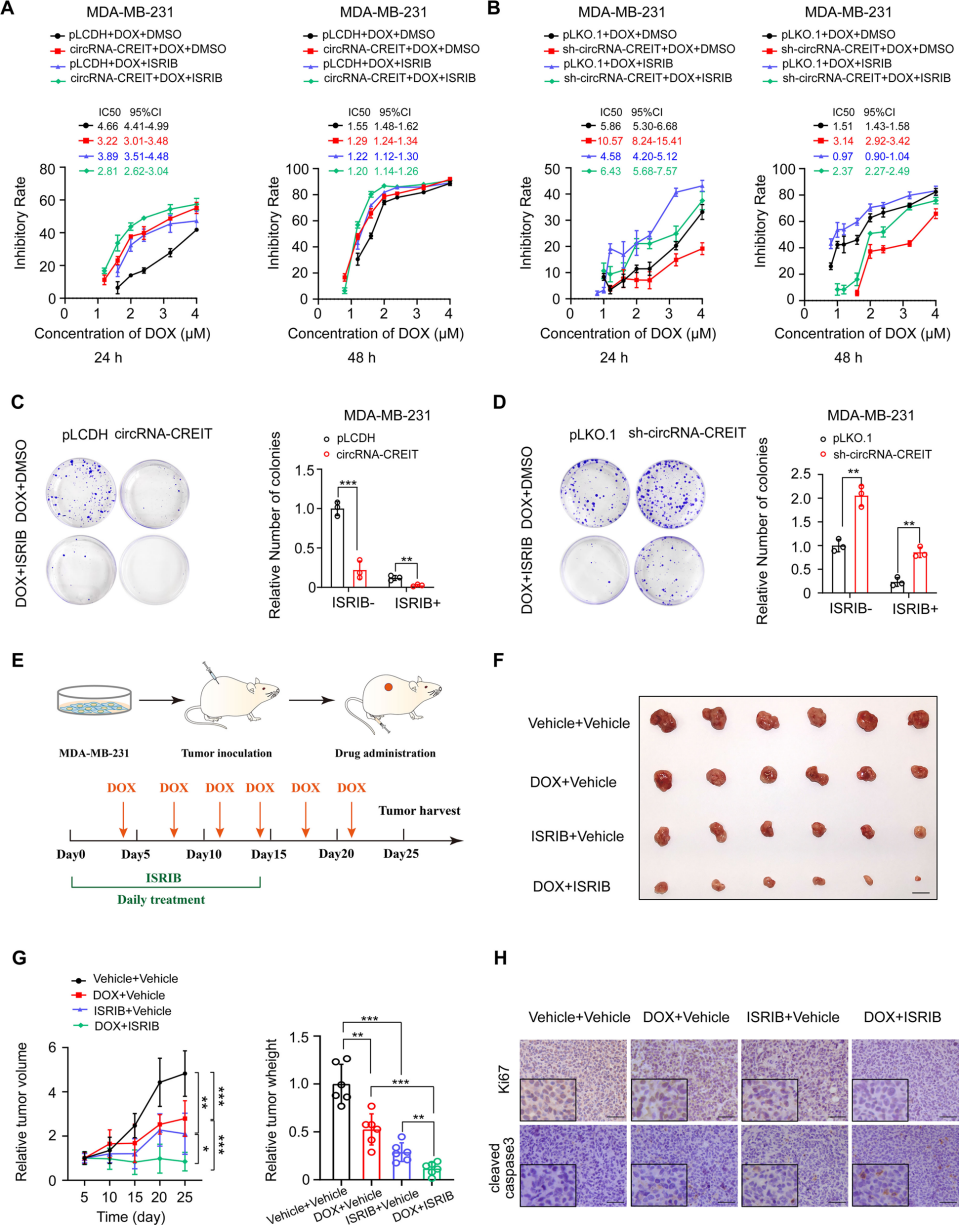
ISRIB exerted synergistic roles with circRNA-CREIT in improving chemosensitivity of TNBC cells. **A** Chemosensitivity to DOX of MDA-MB-231 cells treated with circRNA-CREIT, ISRIB alone or in combination. Cell viability was detected with MTT assays. The IC50 and the 95% CI of cells with different treatments are shown. **B** Chemosensitivity to doxorubicin of MDA-MB-231 cells treated with sh-circRNA-CREIT, ISRIB alone or in combination was detected by MTT assays. The IC50 and the 95% CI of cells with different treatments are shown. **C**, **D** Colony formation assays showed the synergistic roles of circRNA-CREIT and ISRIB in increasing cell chemosensitivity. **E** A schematic diagram indicating the experimental process of constructing the subcutaneous xenograft model and drug administration in female nude mice. **F** Images of xenograft tumors after the indicated treatment. Scale bars = 10 mm. **G** Growth curves and relative weights of the xenograft tumors in the four groups. The group treated with DMSO was used as a control. **H** IHC staining for Ki67 and cleaved caspase-3 expression in the xenograft tumors of different groups. Scale bars = 100 μm. Three independent experiments were conducted for each result. *p < 0.05; **p < 0.01, ***p < 0.001 compared with the controls

It has been well established that the small molecule ISRIB can efficiently reverse the effects of eIF2α phosphorylation and trigger SG disassembly [19]. Our study showed that the combined utilization of the circRNA-CREIT overexpression vector and ISRIB could play a synergistic role and further improve the drug sensitivity of TNBC cells (Fig. 7A). On the other hand, ISRIB notably rescued the chemoresistance caused by circRNA-CREIT knockdown (Fig. 7B). The colony formation assays further corroborated these results (Fig. 7C, D). The synergistic function of circRNA-CREIT and ISRIB in reversing DOX resistance was also confirmed using DOX-resistant cells (Additional file 1: Fig. S11A, B).

To examine the role of ISRIB in chemoresistance in vivo, we subcutaneously injected MDA-MB-231 cells into female nude mice (Fig. 7E). Treatment with DOX alone or ISRIB alone could inhibit the tumor growth in vivo, while the combination of DOX and ISRIB had a dramatic synergistic effect on attenuating tumor growth (Fig. 7F, G). IHC staining for Ki67 and cleaved caspase-3 also validated the above findings (Fig. 7H). The results suggest that ISRIB is a promising drug that could be used in combination with chemotherapy to treat TNBC.

### Exosomal circRNA-CREIT endowed TNBC cells with improved chemosensitivity and a decreased proliferation rate

**Fig. 8:**
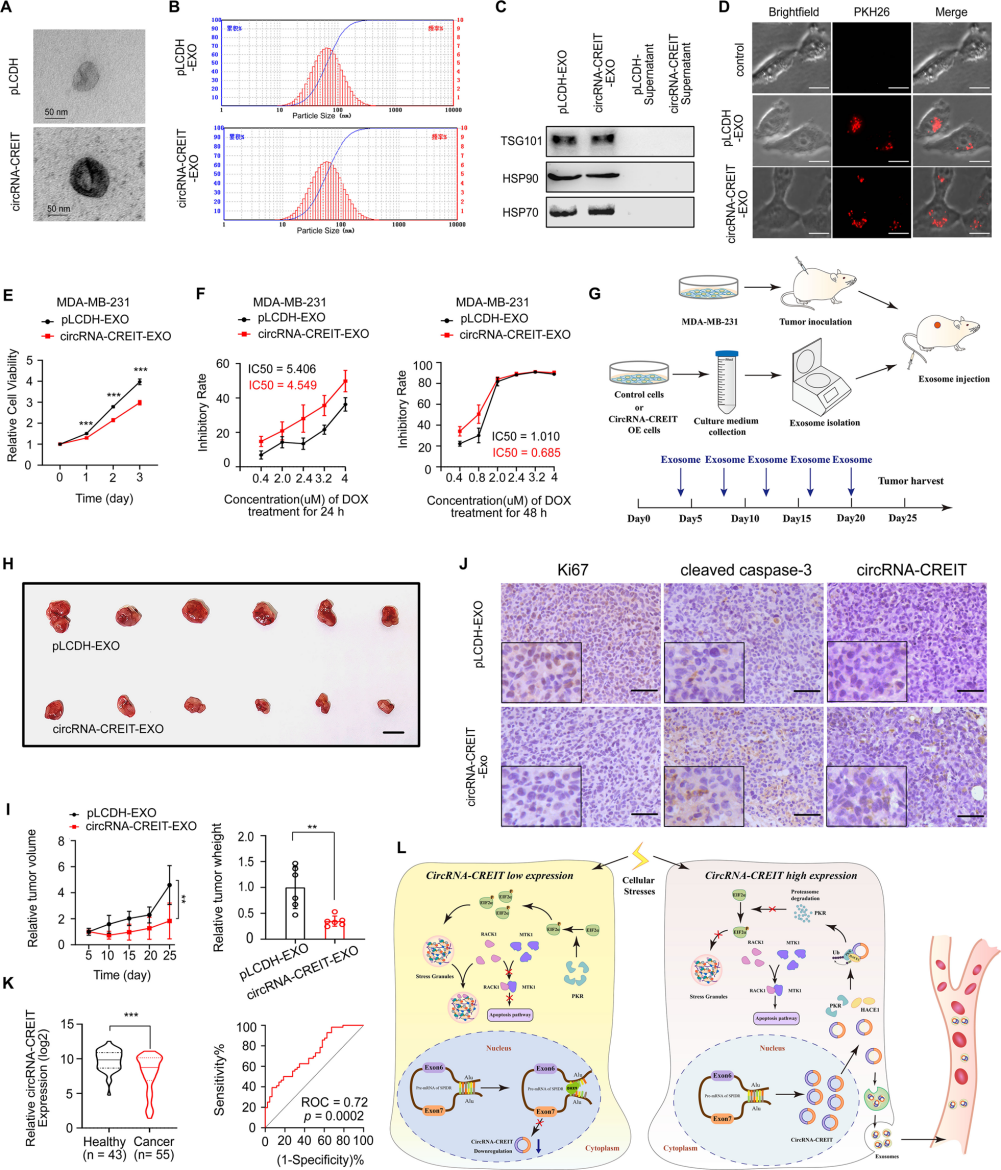
CircRNA-CREIT exerted tumor suppressive roles by exosome transmission in TNBC. **A** Representative images of isolated exosomes analyzed by transmission electron microscopy. **B** The size distribution of the exosomes was measured by a nanolaser particle detector. **C** Western blotting analysis for classic protein markers of exosomes. **D** Representative images of PKH26-stained exosomes that were taken up by TNBC cells. **E** MDA-MB-231 cells were treated with circRNA-CREIT-EXOs or pLCDH-EXOs, and cell viability was examined by MTT assays. **F** MDA-MB-231 cells treated with circRNA-CREIT-exosomes or pLCDH-exosomes were exposed to increasing concentrations of doxorubicin. Cell viability was detected by MTT assays. **G** A schematic diagram indicating the experimental process of constructing the subcutaneous xenograft model and exosome treatment in female nude mice. **H** Photographs of xenograft tumors treated with circRNA-CREIT-EXOs or pLCDH-EXOs. Scale bars = 10 mm. **I** Growth curves (left) and tumor weights (right) of the xenograft tumors. The group treated with exosomes extracted from MDA-MB-231/pLCDH cells served as the control. **J** IHC staining for Ki67 and cleaved caspase-3, and ISH assay for circRNA-CREIT in xenograft tumors treated with exosomes. Scale bars = 100 μm. **K** Violin plot showing the expression of circRNA-CREIT in the plasma of female breast cancer patients and age matched female healthy donors (left). The ROC curve shows that the expression level of circRNA-CREIT could distinguish breast cancer patients from healthy people (right). **L** A schematic diagram shows that circRNA-CREIT suppresses TNBC chemoresistance by inhibiting the formation of SGs. Three independent experiments were conducted for each result. **p < 0.01, ***p < 0.001 compared with the controls

Recent studies demonstrated that circular RNAs could be packaged into exosomes and transmit drug resistance to other cells by exosomes [33]. To test the possibility that circRNA-CREIT could function via exosome transmission, we isolated exosomes from MDA-MB-231 cell culture media with or without circRNA-CREIT overexpression. The morphologies and size distributions of the exosomes were validated with a transmission electron microscope and nanolaser particle detector (Fig. 8A, B). The well-known markers of exosomes were validated by Western blotting (Fig. 8C). As determined by PKH26 staining, PKH26 labeled exosomes could be taken up by MDA-MB-231 cells (Fig. 8D). In the cells treated with circRNA-CREIT-exosomes, significantly suppressed cell proliferation and improved chemosensitivity to DOX were observed (Fig. 8E, F). In addition, the phosphorylation level of eIF2α was downregulated by circRNA-CREIT-exosomes and SG formation was attenuated (Additional file 1: Fig. S12A, B). We next examined the therapeutic effect of circRNA-CREIT-exosomes in vivo (Fig. 8G). The xenograft tumors in the circRNA-CREIT-exosome treated group exhibited a markedly lower growth rate, consistent with the IHC staining for Ki67 and cleaved caspase-3, and the ISH assay confirmed the higher level of circRNA-CREIT compared to the control group (Fig. 8H–J). Additionally, our results revealed that the expression levels of circRNA-CREIT in the plasma of breast cancer patients were significantly lower than those in healthy people, with an area under the ROC (receiver operating characteristic) curve of 0.72 (Fig. 8K). The above data suggest that the circRNA-CREIT-exosome is an effective strategy to treat TNBC and circRNA-CREIT can act as a novel biomarker for breast cancer diagnosis.

## Discussion

As a novel class of transcripts, circRNAs derived from the back-splicing of host genes have gained much attention in recent years [34]. With the advance of high-throughput sequencing technology, increasing numbers of circRNAs have been identified and our understanding of circRNAs has been widely extended. Accumulating evidence has confirmed the important roles of aberrant circRNA expression in cancer development. TNBC is considered one of the most lethal subtypes of breast cancer. The main treatment mode for TNBC is limited to cytotoxic chemotherapy due to the lack of effective therapeutic targets [6]. Doxorubicin (DOX), an anthracycline antibiotic with potent antitumor effects, is commonly employed alone or in combination with other chemotherapeutic agents in the first-line treatment of TNBC [25]. Despite the reasonable effectiveness of DOX in the initial stage of treatment, a large proportion of TNBC patients developed drug resistance and the cancer cells could become more aggressive and refractory [35]. Therefore, it is of great significance to determine the mechanisms of DOX resistance and explore novel strategies to overcome chemoresistance and enhance DOX-based treatment. The functions and molecular mechanisms of circRNAs in TNBC chemoresistance remain largely unclarified.

In the present study, we found that circRNA-CREIT (hsa_circ_0001798) was significantly downregulated in breast cancer, especially in the chemoresistant breast cancer cells and the TNBC subtype. CircRNA-CREIT is derived from exons 6 and 7 of the *SPIDR* gene, which was recently reported to act as a tumor suppressor in cervical adenocarcinoma [36]. However, the potential role of circRNA-CREIT in other types of malignancies is unclear. Our results proved that the RBP DHX9 was an important regulator of circRNA-CREIT biogenesis, which led to the downregulation of circRNA-CREIT. Restoration of circRNA-CREIT expression could significantly enhance the DOX sensitivity of TNBC in vitro and in vivo. A lower expression level of circRNA-CREIT was also related to a higher grade, more lymphatic metastasis, a larger tumor size and a worse prognosis of breast cancer patients. Additionally, circRNA-CREIT was capable of suppressing cell proliferation and migration and promoting cell apoptosis of TNBC cells, which indicated that circRNA-CREIT acted as a robust tumor suppressor. Additionally, we found that circRNA-CREIT could be packaged into exosomes and it conferred increased chemosensitivity to TNBC cells by exosome communication. In addition, circRNA-CREIT manifested higher expression levels in the plasma of breast cancer patients than in healthy people. Therefore, our findings suggest that circRNA-CREIT is also a promising biomarker for breast cancer diagnosis.

The mechanisms of circRNA functions largely depend on the subcellular locations. When located in the nucleus, circRNAs usually regulate gene expression by influencing transcriptional activation [37] or splicing events [38]. However, cytoplasmic circRNAs mainly function by sponging miRNAs or interacting with proteins. In recent years, the miRNA-sponge theory of circRNA has been questioned due to its low abundance and limited miRNA binding sites in most cases [39]. CircRNAs that bind with proteins have attracted much interest, usually acting as protein sponges or scaffolds of protein complexes [30, 31]. For instance, Zhu et al. reported that the cytoplasmic circRNA circZKSCSN1 could suppress the malignant behavior of hepatocellular carcinoma by competitively binding with the RBP FMRP, thereby interfering with the interaction between FMRP protein and CCAR1 mRNAs [40]. Specific secondary and tertiary structures are crucial for circRNA-protein interactions [41]. Du et al. proved that circFoxo3 could interact with the C-terminal RING-finger of MDM2 and the C-terminal regulatory domain of p53, thereby serving as a scaffold to promote the interaction between the two proteins and subsequently enhancing cell apoptosis [41]. In the present study, circRNA-CREIT was predominantly located in the cytoplasm and PKR was identified as an interactor of circRNA-CREIT by RNA pulldown, MS and RIP assays. The typical regulators of PKR are dsRNAs, such as viral RNAs and endogenous RNAs transcribed from genomic repeat elements [42]. Apart from dsRNAs, cellular RNAs with secondary structures can also bind to PKR and act as PKR regulators [43]. For instance, linc00665 promoted PKR activation by directly binding with the PKR protein and enhancing its stability [44]. PKR possesses two N-terminal dsRNA (double-stranded RNA) binding motifs (dsRBM1 and dsRBM2) and one C-terminal kinase domain. To map which domain mediated the interaction between circRNA-CREIT and PKR, we constructed truncates based on the secondary structures, respectively. We found that stem loop #2 of circRNA-CREIT mediated binding with dsRBMs of the PKR protein. Additionally, it was worth noting that circRNA-CREIT and PKR protein also showed colocalization in the nucleolus of TNBC cells (Fig. 3F). According to Holdt et al., circRNAs located in the nucleolus could modulate the process of ribosomal RNA maturation [45]. Besides, the location of PKR proteins in nucleolus has been previously reported [46], which is intimately associated with the development and progression of human cancers [47]. Therefore, we speculate that the interaction between circRNA-CREIT and PKR may regulate the functions of nucleolus, which needs further investigations. In the current study, we focused on the functions of circRNA-CREIT and PKR in the cytoplasm.

Ubiquitination is one of the most common and important posttranslational modifications of proteins [48]. Different types of ubiquitination mediate distinct fates of substrate proteins. For instance, K48-linked polyubiquitination usually leads to proteasome-dependent degradation of proteins, while K63-linked polyubiquitination is implicated in signaling transduction [48]. CircRNAs play a critical role in regulating the protein stability and functions by affecting the ubiquitination process. CircRNA-SORE, which is significantly associated with sorafenib resistance in hepatocellular carcinoma, could bind with the oncogenic protein YBX1 in the cytoplasm and inhibit its interaction with the E3 ubiquitin ligase PRP19, thereby suppressing ubiquitin–proteasome-mediated degradation of YBX1 [33]. In addition, Li et al. demonstrated that circNDUFB2 served as a scaffold for IGF2BPs and the E3 ligase TRIM25 to enhance their interactions, thus leading to an increase in IGF2BP ubiquitination and degradation in lung cancer cells [49]. Here, we demonstrated that circRNA-CREIT acted as a scaffold to increase the E3 ligase HACE1-mediated K48-linked ubiquitination and degradation of PKR proteins. Our data provide a novel mechanism for PKR regulation and sound evidence for circRNA engaging in protein metabolism.

PKR, namely EIF2AK2, a member of the eukaryotic initiation factor-2 subunit α (EIF2α) kinase family, initially became well-known due to its antiviral roles [50]. PKR can be activated in response to multiple cellular stresses, such as viral infection, cytotoxic cytokines, DNA damage and oxidative stress [51]. eIF2α is a well-studied and important executor of PKR downstream signals under stress. It should be noted that most PKR-triggered signaling pathways are two-faced and PKR regulates cell fates in a context-dependent manner [42]. This makes sense because the roles of PKR in cancer development and progression are controversial. For instance, phosphorylation of eIF2α induced by PKR activation could trigger stress granule formation under arsenic trioxide treatment, which granted stem cell properties and chemoresistance in refractory glioblastoma [32]. Another study demonstrated that knocking down PKR or using PKR inhibitors suppressed the growth of APC-mutated colorectal cancer which favored the oncogenic role of PKR [52]. However, Pataer et al. reported that PKR/eIF2α activation mediated the apoptosis induced by Ad-mda7 overexpression in lung cancer, indicating that the PKR/eIF2α axis could act as a tumor suppressor in some cases [53]. In breast cancer, increased expression of PKR was reported [54, 55], and loss of PKR contributed to reduced cell viability of MCF-7 and MDA-MB-231 breast cancer cells [55]. Consistently, our data demonstrated the oncogenic roles of PKR and showed that PKR overexpression could reverse the effect of circRNA-CREIT on increasing chemosensitivity in TNBC.

Triggering SG formation is an important effect of the PKR/eIF2α signaling axis. In recent years, a close association between SGs and chemoresistance has been gradually revealed [13]. SGs are cytosolic membraneless organelles mainly composed of untranslated mRNAs and their associated proteins. Phase separation and condensation of the molecular components are considered to mediate the formation of SGs [56]. When the cells are exposed to environmental stresses, such as arsenite, hypoxia, heat shock and chemotherapeutic drugs, eIF2α can be phosphorylated by stress-sensing serine/threonine kinases at serine residue 51. Phosphorylated eIF2α inhibits its efficient GDP-GTP exchange, leading to depletion of the eIF2α/GTP/tRNAi^Met^ ternary complex and arrest of translation initiation [11]. To protect untranslated mRNAs from degradation, assembly of SGs occurs and polysome-released mRNAs are recruited into SGs [57]. Many proteins are also recruited to SGs, including translation initiation factors and other proteins related to SG assembly or functions [58]. For instance, sequestration of RACK1 protein by SGs could block its binding with MTK1, thus preventing the activation of the MTK1-mediated stress-responsive apoptosis pathway [13]. After the withdrawal of cellular stresses, SGs are able to disassemble and release the mRNAs for translation [59]. Therefore, SGs are dynamic cellular structures that favor cell survival under harmful conditions and contribute to chemoresistance in cancer. In our present study, we demonstrated that circRNA-CREIT could inhibit DOX-induced SG formation by attenuating the phosphorylation of eIF2α via PKR. We also showed that the interaction between RACK1 and MTK1 might mediate the functions of circRNA-CREIT. Importantly, our data demonstrated that the small molecule ISRIB, which could reverse the effect of phosphorylated eIF2α and SG formation, exhibited excellent synergistic therapeutic effects with chemotherapy in TNBC. The curative effects of ISRIB have been reported in prostate cancer [60] and acute myeloid leukemia [61]. However, the function of ISRIB in breast cancer is rarely reported and we are the first to reveal the effect of ISRIB on reversing doxorubicin resistance in TNBC.

## Conclusions

In summary, our study revealed the crucial roles of circRNA-CREIT in regulating SG formation and TNBC chemoresistance. CircRNA-CREIT enhanced HACE1-mediated PKR degradation through the ubiquitin–proteasome system, thereby inhibiting the PKR/eIF2α signaling axis and SG formation. Additionally, circRNA-CREIT could be transmitted by exosomes and disseminate chemosensitivity among TNBC cells. Clinically, circRNA-CREIT was significantly downregulated in TNBC tissues and plasma from breast cancer patients and high expression of circRNA-CREIT was closely associated with favorable prognosis. In addition, the biogenesis of circRNA-CREIT was downregulated by DHX9 (Fig. 8L). Taken together, our study demonstrated that circRNA-CREIT is a potential biomarker for breast cancer diagnosis and prognosis. Targeting circRNA-CREIT and SG formation could be effective strategies for enhancing TNBC chemosensitivity.

## Supplementary Information

**Additional file 1**. Supplementary Materials and Methods, Supplementary Tables, and Supplementary Figures.

## Data Availability

All data are available in the main text or Additional file 1.

## Abbreviations

TNBC: Triple-negative breast cancer
ER: Estrogen receptor
PR: Progesterone receptor
HER-2: Human epidermal growth factor
circRNA: Circular RNA
TCGA: The Cancer Genome Atlas
ISH: In situ hybridization
FISH: Fluorescence in situ hybridization
RIP: RNA immunoprecipitation
co-IP: Co-immunoprecipitation
SG: Stress granules
