# Supplementary material for: CircRNA-CREIT inhibits stress granule assembly and overcomes doxorubicin resistance in TNBC by destabilizing PKR
Source: J Hematol Oncol. 2022 Aug 29;15:122. doi: 10.1186/s13045-022-01345-w (PMC9425971; doi:10.1186/s13045-022-01345-w)
Supplement: Supplementary file 1 — Additional file 1. Supplementary Materials and Methods, Supplementary Tables, and Supplementary Figures. [file 13045_2022_1345_MOESM1_ESM.docx]

**Supplementary Materials**

**Supplementary Materials and Methods**

**Nucleic Acids extraction and Quantitative real-time PCR (qRT-PCR)**

Total RNAs of whole cell lysates were isolated using the RNA-easy Isolation Reagent (Vazyme, Nanjing, China). The purity and concentration of extracted RNAs were measured on a NanoDrop 2000 (Thermo Fisher Scientific, Waltham, USA). 500 ng of total RNAs was subjected to synthesize cDNA with the PrimeScript reverse transcriptase reagent kit (Takara, Shiga, Japan). Then qRT-PCR was conducted on the Light Cycler 480 II Real-Time PCR System (Roche, Switzerland) using SYBR Premix Ex TaqII (Takara) to determine the RNA levels indicated. GAPDH was used as internal reference and qRT-PCR primer sequences were provided in Supplementary Table 5. The PARIS™ Kit (Invitrogen) was applied to separation of cytoplasmic and nuclear RNAs. The genomic DNA was extracted with the TIANGEN Genomic DNA Kit (TIANGEN, Beijing, China).

**RNA sequencing (RNA-seq)**

To screen differentially expressed circRNAs in breast cancer, ribosomal RNA-depleted total RNA sequencing was performed using 6 pairs of breast cancer tissues and adjacent normal mammary tissues. The total RNA of the tissues was extracted using the RNA-easy Isolation Reagent (Vazyme, Nanjing, China), as described above. The concentration, purity and quality of RNAs were examined by a NanoDrop 2000 (Thermo Fisher Scientific, Waltham, USA) and RNA electrophoresis. After depleting the ribosomal RNA, the cDNA libraries were constructed and RNA-seq was performed on the Illumina sequencing platform by LC-BIO Co., Ltd (HangZhou, China). R package “edgeR” was utilized to explore differentially expressed circRNAs with the criteria of fold change > 1.5 and *p* value < 0.05. GraphPad Prism V8.3.0 was used to draw the corresponding volcanic plot and the circos plot was generated by the Strawberry Perl (version-5.32.1.1) software.

**Protein extraction, Western blotting, immunoprecipitation (IP) assay and antibodies**

Total protein of cells was extracted with the Western and IP lysis buffer (Beyotime, Shanghai, China) supplemented with the protease inhibitors. The concentration of the proteins was detected using the BCA Protein Assay Kit (Millipore, Burlington MA, USA).

For Western blotting assay, extracted total proteins were separated by SDA-PAGE gel electrophoresis and transferred to the polyvinylidene fluoride (PVDF) membrane (Millipore, Burlington MA, USA). Then the PVDF membrane was blocked with 5% skimmed milk for an hour and incubated with primary antibodies overnight at 4 ℃. After incubation with the secondary antibodies, the signal of relevant proteins was detected with the ECL chemiluminescence detection kit (Vazyme, Nanjing, China). For IP assay, the protein extracted was precleared with protein A/G magnetic bead (Bimake, Texas, USA), followed by incubation with indicated primary antibodies for 2 hours at 4 ℃. Then the protein A/G magnetic bead was added into the system. After incubation at 4 ℃ overnight, the supernatant was discarded and the beads were collected with a magnetic separator. Then the beads were washed five times with TBST buffer and the proteins bound to the beads were dissolved.

The antibodies used in the study were as follows: anti-PKR (ImmunoWay Biotechnology, Texas, USA), anti-MYC (ImmunoWay Biotechnology, Texas, USA), anti-p-eIF2α (Ser51) (ImmunoWay Biotechnology, Texas, USA), anti-EIF3A (ImmunoWay Biotechnology, Texas, USA), anti-EIF4G1 (ImmunoWay Biotechnology, Texas, USA), anti-G3BP1 (ImmunoWay Biotechnology, Texas, USA), anti-G3BP2 (ImmunoWay Biotechnology, Texas, USA), anti-HSP90 (ImmunoWay Biotechnology, Texas, USA), anti-HACE1 (Abcam, Cambridge, UK), anti-HSP70 (Abcam, Cambridge, UK), anti-MTK1 (Abcam, Cambridge, UK), anti-cleaved caspase-3 (Cell Signaling Technology, MA, USA), anti-caspase-3 (Cell Signaling Technology, MA, USA), anti-cleaved caspase-7 (Cell Signaling Technology, MA, USA), anti-caspase-7 (Cell Signaling Technology, MA, USA), anti-cleaved PARP (Cell Signaling Technology, MA, USA), anti-PARP (Cell Signaling Technology, MA, USA), anti-FLAG (Invitrogen, Texas, USA), and anti-β-actin (Sigma-Aldrich, MO, USA).

**Immunohistochemistry (IHC) assay**

Formalin fixed tumor tissues were dehydrated and embedded in paraffin, and then cut into sections. The sections were deparaffinized by xylene and rehydrated through successive incubation with 100%, 95%, 80% and 70% ethanol solutions. After antigen retrieval with sodium citrate or EDTA solutions, the endogenous peroxidase and nonspecific binding sites were blocked with 1% H_2_O_2_ and goat serum, respectively. Then, the slides were incubated with indicated primary antibodies at 4 ℃ for 12-16 hours. The next day, after washed with PBS buffer, the sections were successively incubated with biotinylated secondary antibodies and streptavidin-conjugated horseradish peroxidase (HRP). The HRP signal was detected with DAB chromogenic kit (ZSGB-BIO, Beijing, China) and hematoxylin was used to distinguish nucleus. Lastly, the slides were dehydrated through successive incubation with 70%, 80%, 95%, 100% ethanol solutions, and cleared in xylene. Leica light microscope was utilized for observation and photo capture.

**Plasmids construction and transfection**

Human circRNA-CREIT overexpressing vector pLCDH-circRNA-CREIT and control plasmid pLCDH-ciR were purchased from Geneseed Biotechnology Company (Guangzhou, China). To generate overexpression vectors of PKR or RACK1, the coding sequence of human PKR or RACK1 was cloned into the pcDNA3.1 plasmid, respectively. pEnter plasmid was used to construct HACE1 overexpression vectors. The MTK1 overexpression plasmid pHAGE-MTK1 was obtained from Addgene platform(*1*). To observe the subcellular location of RACK1 proteins, the coding sequence of RACK1 was constructed into pmCherry-C1 vectors. Short-hairpin RNAs (shRNAs) against the back-splicing site of circRNA-CREIT were constructed into pLKO.1-puro vectors. The targeted sequences of shRNAs were listed in Supplementary Table 3. Lipofectamine 2000 (Invitrogen, CA, USA) was used in the plasmid transfection and stable cell lines were constructed by puromycin or neomycin treatment.

**(3-(4,5-dimethyl-2-thiazolyl)-2,5–diphenyl-2H-tetrazolium bromide) MTT and colony formation assay**

Cell proliferation rate and chemosensitivity were assessed by MTT assay. Briefly, TNBC cells were seeded into 96-well cell culture plates at a density of 2000 cells/well for proliferation assay and 4000 cells/well for IC50 (half-maximal inhibitory concentration) assessment. For IC50 assay, cells were treated with indicated drugs or DMSO as control after cell attachment and incubated for indicated time. Then, 20 μl of MTT (5 mg/ml) was added to the cells. After incubation for another 4 hours at 37 ℃, the supernatants were aspirated and the formazan was dissolved by 100 μl DMSO per well. The absorbance values were measured by a microplate reader (Bio-Rad, CA, USA) at 490 nm.

Colony formation assay was also performed for assessment of cell viability. 1500 TNBC cells with indicated treatment were inoculated into a 60 mm cell culture disk. After incubation for 14 days, the cells were washed with PBS, fixed by methanol for 5 min and stained with 0.2% crystal violet for 15 min.

**Transwell and wound healing assay**

Cell culture inserts with transparent PET membrane (8 mm pore size, 24 wells, Corning, NY, USA) was used for migration and invasion assays. For migration assay, 1×10^5^ TNBC cells were resuspended by DMEM without serum and seeded into the upper chambers of 24-well plates. 600 μL of FBS containing cell culture medium was added into the lower chamber. For invasion assay, 1×10^5^ TNBC cells were inoculated in the upper chamber of Matrigel-coated inserts. After incubation for 24-48 h at 37 °C, cells were washed with PBS, fixed by methanol and stained with 0.2% crystal violet. Cells that did not penetrate the membrane were carefully wiped off with a cotton swab.

For wound healing assay, TNBC cells were seeded in 24 well plates at the density of 2× 10^5^ cells per well. After incubation for 24 h at 37 °C, the streaks of cell monolayer were generated by scratching with a 10 μL pipette tip. Then the floating cells were washed with PBS. After indicated time, the cells were photographed under an Olympus light microscope (Olympus, Tokyo, Japan).

**RNA pulldown assay and MS analysis**

Pierce Magnetic RNA-Protein Pull-Down Kit (ThermoFisher, MA, USA) was used to detect the interaction between circRNA-CREIT and indicated proteins. The experiments were conducted according to the manufacturer’s protocols. Briefly, Cell lysates of 2×10^6^ cells were prepared using Western and IP cell lysis buffer (Beyotime) supplemented with the protease inhibitor cocktail (Cell Signaling Technology). Biotin-labeled circRNA probes and control probes were incubated with washed streptavidin magnetic beads at room temperature for 30 min with agitation. Next, the streptavidin magnetic beads with immobilized probes were incubated with prepared cell lysates at 4 °C for 60 min with rotation. The magnetic beads were then washed and the proteins pulled down were eluted. Indicated proteins were identified by Western blotting and Mass spectrometry (MS) analysis. For MS analysis, protein samples obtained from RNA pull-down were firstly separated by SDS-PAGE gel electrophoresis and stained with Coomassie brilliant blue. Then the gel strips were cut off and sent to PTMBIO company (Hangzhou, China) for protein identification by MS analysis.

**RNA immunoprecipitation (RIP) assay**

RIP assay was performed using the Magna RIP RNA-Binding Protein Immunoprecipitation Kit (Millipore, MA, USA). In brief, approximate 1×10^7^ cells were lysed in RIP lysis buffer and stored at -80 °C. Specific antibodies were incubated with magnetic beads with rotation for 30 min at room temperature. Then the cell lysates were thawed quickly and centrifuged at 14, 000 rpm for 10 min at 4 °C. The supernatant was collected and immunoprecipitated with antibody-coated beads overnight at 4 °C. The next day, the beads were washed six times and the coprecipitated RNAs were purified. qRT-PCR was carried out to detect the enrichment of circRNA-CREIT in different groups.

**Mouse xenograft studies**

Female BALB/c nude mice (4-5weeks) were used in animal studies (Vital River Laboratory Animal Technology, Beijing, China). For the mouse model that examined the effect of circRNA-CREIT overexpression on chemosensitivity, twenty BALB/c nude mice were randomly divided into four groups (group A, B, C and D). Mice in group A and group C were subcutaneously injected with MDA-MB-231 cells with pLCDH-circRNA-CREIT stable overexpression; in group B and group D, mice were injected with control cells MDA-MB-231/pLCDH-ciR. When the xenografts reached about 100 mm^3^, mice in group C and D was treated with doxorubicin via intraperitoneal injection (2 mg/kg/mouse, twice a week for 3 weeks), and mice in group A and B was treated with vehicle control. The tumor size was recorded every five days by measuring the length (L) and width (W) with a caliper. The tumor volume was calculated as L × W^2^/2. After treatment, all the mice were sacrificed and the xenograft tumors were removed for further studies. *In vivo* study of the effect of circRNA-CREIT knockdown on chemosensitivity was similar to the experiments described above.

For the experiments that verified the roles of ISRIB in enhancing doxorubicin chemosensitivity, 24 female BALB/c nude mice (4-5 weeks) were randomly assigned to four groups (a, b, c and d). All the mice were subcutaneously injected with MDA-MB-231 cells. After the tumor volume reached about 100 mm^3^, mice of four groups were treated with doxorubicin (2 mg/kg/mouse, twice a week for 3 weeks), ISRIB (2.5 mg/kg/mouse, daily for 2 weeks), DMSO alone or in combination, respectively. The measurement of tumor size was as described above.

For exosome related *in vivo* studies, 12 female BALB/c nude mice (4-5 weeks) were randomly divided into two groups. All the mice were subcutaneously injected with MDA-MB-231 cells. When the volume of xenografts reached 100 mm^3^, 30 μg exosomes extracted from circRNA-CREIT overexpressing cells or control cells were injected to mice in the two groups via tail vein, respectively (every 4 days for 3 weeks). The measurement of tumor size was as described above.

All in vivo studies were approved by the Animal Care and Use Committee of Shandong University and conducted in accordance with the guidelines for animal experiments. No blinding was performed in our animal experiments.

**Exosome extraction and Exosome uptake assay**

Exosomes from circRNA-CREIT overexpressing MDA-MB-231 and control cells were isolated by ultracentrifugation. Briefly, TNBC cells were cultured in exosome-free complete medium and the supernatants were collected. Through successive centrifugation at 300 × g for 10 min, 2000 ×g for 10 min, 10,000 × g for 70 min and 110,000 × g for 70 min, cell debris and other larger vesicles were removed while exosomes were collected. After being washed with PBS and re-collected by ultra-centrifugation, the exosomes were resuspended in PBS and filtered through 0.22 μm filters (Millipore, Burlington MA, USA). The morphology of isolated exosomes was verified under a transmission electron microscope (Hitachi HT7700, Tokyo, Japan), and the size distribution was measured by a nano-laser particle detector (Winner, Guangdong, China). Western blotting was used to detect the expression of exosome markers. For exosome uptake assay, PKH26 (Sigma-Aldrich, MO, USA) was used to label the exosome membrane. The exosomes were washed twice with PBS and the redundant dye was removed by ultra-centrifugation. After PKH26-labeled exosomes were added into cells for 12 hours, the uptake of exosomes was observed under a fluorescence microscope (Olympus, Tokyo, Japan).

**RNA fluorescence in situ hybridization (FISH) assay**

Cy3-labeled probes targeting the splicing junction of circRNA-CREIT was designed. The targeted sequence of which was shown in Supplementary Table 3. FISH assay was performed with the RNA FISH Kit according to the manufacturer’s protocols (GenePharma, Jiangsu, China). The subcellular location of circRNA-CREIT was observed and photographed under a fluorescence microscope (Leica, Wetzlar, Germany).

**Immunofluorescence assay**

Approximate 6×10^4^ cells were seeded onto glass slides placed in 24 well plates and cultured for 24 h at 37 ℃. The next day, the cells were washed three times with PBS and fixed with 4% paraformaldehyde for 15 min. Then cells were permeabilized with 0.3% Triton X-100 for 25 min and blocked with 10% goat serum for an hour, followed by incubation with indicated primary antibodies overnight at 4 ℃. The next day, the cells were incubated with fluorescent secondary antibodies (ZSGB-BIO, Beijing, China) at room temperature in dark for 1 h. After three washes, the nucleus was stained with DAPI. Images were acquired with a fluorescence microscope (Leica, Wetzlar, Germany).

**Flow cytometry analysis**

The apoptotic levels of cells were detected by Flow cytometry analysis using the BD PE Annexin V Apoptosis Detection Kit (BD Biosciences, NJ, USA). Upon indicated treatments, cells were digested with trypsin and washed twice with PBS. Then the cells were resuspended in the binding buffer provided in the kit and stained with proper dyes. After incubation for 15min in dark at room temperature, the apoptotic rate of cells was analyzed using a FACScan flow cytometry (BD Biosciences, NJ, USA).

**Human breast cancer** **organoids and treatments**

Fresh specimens of breast cancer tissues were cut into 1mm^3^ size fragments on ice and then digested for 2 h at 37 °C with gentle shake. The digestion medium was DMEM/F12 (Macgene, Beijing, China) supplemented with Y-27632 (5 μM, MCE, NJ, USA), Primocin (Invivogen, Toulouse, France), 1% BSA, ITS-G (BasalMedia Technologies Company, Shanghai, China), HEPES (10mM, Thermo Fisher Scientific, MA, USA), hyaluronidase (1000 U/mL, Sigma-Aldrich, MO, USA), collagenase I and III (300 U/mL in total, Worthington, Milano, Italy) and DNase I (10mg/ml). After digestion, the tissues were filtered with a 100 μm filter strainer and centrifuged at 300 g for 5 min at 4 °C. Then the sediment was resuspended with digestion termination solution (DMEM/F12 supplemented with 0.1% BSA and Primocin) and centrifuged again. Next TAC buffer was used to lyse the erythrocytes by resuspending the sediment. Then the cell pellet was washed twice with the digestion termination solution and centrifuged again at 300 g for 5 min at 4 °C. The cell pellet was resuspended by the growth factor reduced Matrigel (R&D Systems, MN, USA) and seeded into 48-well tissue culture plates. The gel was allowed to polymerize for 60 min at 37 °C and then overlaid with 300 μl organoid culture medium. The organoid medium was DMEM/F12 containing 10 mM HEPES, 1×B27 (Gibco, CA, USA), 5 ng/ml recombinant human EGF (PeproTech, NJ, USA), 5 ng/ml FGF7 (PeproTech, NJ, USA), 20 ng/ml FGF10 (PeproTech, NJ, USA), 1×GlutaMax (Invitrogen, Texas, USA), 1×Primocin, 10 mM Nicotinamide (Sigma-Aldrich, MO, USA), 1.25 mM N-acetyl-L-cysteine (Sigma-Aldrich, MO, USA), 100 ng/mL Noggin (Peprotech, NJ, USA), 5 nM Neuregulin 1 (PeproTech, NJ, USA), 500 nM A83-01 (Tocris Bioscience, MN, USA), 500 ng/mL R-spondin (BioLegend, CA, USA), 5 µM Y-27632 (Sigma-Aldrich, MO, USA) and 10 μM SB202190 (Sigma-Aldrich, MO, USA). The medium was changed every three days. The passage of organoids was used TrypLE (Gibco, CA, USA) for digestion and the organoids were re-seeded in new tissue culture plates. For determining the chemosensitivity of different organoids, the organoids were grown in 96-well plates and treated with different concentration of DOX for 48 h, followed by CCK8 assay for cell viability detection.

**References**

1. P. K. Ng, J. Li, K. J. Jeong, S. Shao, H. Chen, Y. H. Tsang, S. Sengupta, Z. Wang, V. H. Bhavana, R. Tran, S. Soewito, D. C. Minussi, D. Moreno, K. Kong, T. Dogruluk, H. Lu, J. Gao, C. Tokheim, D. C. Zhou, A. M. Johnson, J. Zeng, C. K. M. Ip, Z. Ju, M. Wester, S. Yu, Y. Li, C. P. Vellano, N. Schultz, R. Karchin, L. Ding, Y. Lu, L. W. T. Cheung, K. Chen, K. R. Shaw, F. Meric-Bernstam, K. L. Scott, S. Yi, N. Sahni, H. Liang, G. B. Mills, Systematic Functional Annotation of Somatic Mutations in Cancer. *Cancer cell* **33**, 450-462.e410 (2018).

**Supplementary Tables**

**Supplementary Table 1. The clinicopathological features of**

**patients in cohort 1.**

| **Characteristics** | **Total cases (n = 321)** | **circRNA-CREIT expression level** | | |
| --- | --- | --- | --- | --- |
|  |  | **Low** | **High** | ***p* value** |
| **Age** |  |  |  |  |
| ≤ 50 | 167 | 85 | 82 | 0.5314 |
| > 50 | 154 | 73 | 81 |  |
| **Menopausal state** |  |  |  |  |
| Pre | 142 | 72 | 70 | 0.6359 |
| Post | 179 | 86 | 93 |  |
| **Ki67** |  |  |  |  |
| Low | 91 | 40 | 51 | 0.1422 |
| High | 230 | 122 | 108 |  |
| **Pathological grade** |  |  |  |  |
| ≤ 2 | 225 | 106 | 119 | 0.1352 |
| > 2 | 81 | 46 | 35 |  |
| unknown | 15 | 2 | 13 |  |
| **Lymphatic metastasis** |  |  |  |  |
| Negative | 166 | 71 | 95 | 0.0167* |
| Positive | 155 | 87 | 68 |  |
| **Tumor size** |  |  |  |  |
| ≤ 2 | 143 | 71 | 72 | 0.5902 |
| > 2 | 178 | 83 | 95 |  |
| **ER** |  |  |  |  |
| Positive | 212 | 98 | 114 | 0.2618 |
| Negative | 96 | 51 | 45 |  |
| unknown | 13 | 9 | 4 |  |
| **PR** |  |  |  |  |
| Positive | 199 | 97 | 102 | 0.8837 |
| Negative | 121 | 60 | 61 |  |
| unknown | 1 | 1 | 0 |  |
| **HER-2** |  |  |  |  |
| Positive | 64 | 42 | 22 | 0.0012** |
| Negative | 214 | 91 | 123 |  |
| unknown | 43 | 25 | 18 |  |

***. *p* < 0.05; **. *p* < 0.01.**

**Supplementary Table 2. The clinicopathological features of**

**patients in cohort 2.**

| **Characteristics** | **Total cases (n = 58)** | **patients with chemotherapy** | | |
| --- | --- | --- | --- | --- |
|  |  | **Sensitive** | **Resistant** | ***P* value** |
| **Age** |  |  |  |  |
| ≤50 | 31 | 21 | 10 | 0.5025 |
| >50 | 27 | 16 | 11 |  |
| **Menopausal state** |  |  |  |  |
| Pre | 30 | 19 | 11 | 0.9399 |
| Post | 28 | 18 | 10 |  |
| **Ki67** |  |  |  |  |
| Low | 13 | 10 | 3 | 0.2634 |
| High | 45 | 27 | 18 |  |
| **Pathological grade** |  |  |  |  |
| ≤2 | 30 | 18 | 12 | 0.5338 |
| >2 | 28 | 19 | 9 |  |
| **Lymphatic metastasis** |  |  |  |  |
| Negative | 23 | 14 | 9 | 0.7072 |
| Positive | 35 | 23 | 12 |  |
| **Tumor size** |  |  |  |  |
| ≤2 | 23 | 14 | 9 | 0.7072 |
| >2 | 35 | 23 | 12 |  |
| **ER** |  |  |  |  |
| Positive | 11 | 5 | 6 | 0.1598 |
| Negative | 47 | 32 | 15 |  |
| **PR** |  |  |  |  |
| Positive | 11 | 5 | 6 | 0.1598 |
| Negative | 47 | 32 | 15 |  |
| **HER-2** |  |  |  |  |
| Positive | 9 | 6 | 3 | 0.8453 |
| Negative | 49 | 31 | 18 |  |

**Supplementary Table 3. Sequences for the nucleic acids used in the study.**

| **Targets** | **Targeted Sequence (5’-3’)** |
| --- | --- |
| **Sh1-circRNA-CREIT** | GACACTTTCAGCCAAGTTCTA |
| **Sh2-circRNA-CREIT** | AAAAGACACTTTCAGCCAAGT |
| **Sh3-circRNA-CREIT** | ACTTTCAGCCAAGTTCTATAG |
| **Si-DHX9** | GAGCCAACTTGAAGGATTA |
| **circRNA-CREIT**  **probe (ISH)** | CAAAAGACACTTTCAGCCAAGTTCTATA |
| **circRNA-CREIT**  **probe (FISH)** | ACCAAAAGACACTTTCAGCCAAGTTCTAT |

Supplementary Table 4. Univariate and Multivariate analyses for the overall survival of 244 breast cancer patients with complete data in Qilu hospital.

|  | Univariate analysis ^a^ |  | Multivariate analysis ^b^ |  |
| --- | --- | --- | --- | --- |
| Index | **HR (95% CI)** | ***p* value** | **HR (95% CI)** | ***p* value** |
| Age ^c^ | 0.908 (0.52-1.588) | 0.735 |  |  |
| Menopausal status  (pre vs. post) | 1.276 (0.733-2.223) | 0.389 |  |  |
| Pathological Grade  (> G2 vs. ≤ G2) | 2.07 (1.188-3.607) | 0.01* | 2.043 (1.172-3.563) | 0.012* |
| ER status  (ER+ vs. ER-) | 0.578 (0.331-1.006) | 0.053 |  |  |
| PR status  (PR+ vs. PR-) | 0.835 (0.477-1.461) | 0.527 |  |  |
| HER2 status  (HER2+ vs. HER2-) | 1.033 (0.516-2.066) | 0.927 |  |  |
| Lymph node status  (with metastasis vs. without metastasis) | 1.776 (0.979-3.223) | 0.059 |  |  |
| Ki67 positive rate  (High vs. low) | 0.772 (0.386-1.544) | 0.465 |  |  |
| Tumor size ^c^ | 0.763(0.428-1.36) | 0.359 |  |  |
| circRNA-CREIT  (High expression vs. low expression) | 0.498(0.275-0.903) | 0.022* | 0.505 (0.279-0.916) | 0.024* |

a. Cox's proportional hazards regression model was utilized.

b. Only variables with p < 0.05 in univariate analysis were incorporated into the multivariate Cox proportional hazard regression analysis. HR, hazard ratio; CI, confidence interval.

c. Age and tumor size were considered as continuous variables.

*. p < 0.05.

**Supplementary Table 5. Primer sets used for qRT-PCR**

| **Primer set** | **Primers** | **Sequence (5’-3’)** |
| --- | --- | --- |
| **circRNA-CREIT** | Forward | AGATCTGCTATTTCTTTGTGGAGAC |
|  | Reverse | GAATCAATGAGTAGGACATTTTCCA |
| **SPIDR** | Forward | AGAAAAAGGAGTTGGAATACAGAATG |
|  | Reverse | CGGATTCCCAGAAGTGTTCTG |
| **DHX9** | Forward | CGAACCATCTCAGCGACAAAA |
|  | Reverse | TGAGGTCCATGCTTATTTGCTC |
| **PKR** | Forward | GCCGCTAAACTTGCATATCTTCA |
|  | Reverse | TCACACGTAGTAGCAAAAGAACC |
| **Alu19**  **(RIP assay)** | Forward | CATCCCCTGCTTCCTTTCTTC |
|  | Reverse | GAAGAGTTAAGTGTCAGAAAAGGTCTG |
| **Alu21**  **(RIP assay)** | Forward | ATCAAAAAGAATGAAATTCGGGA |
|  | Reverse | CCTTGACATCCATGATTAAGTTTATTC |
| **U6** | Forward | GGAACGATACAGAGAAGATTAGC |
|  | Reverse | TGGAACGCTTCACGAATTTGCG |
| **β-actin** | Forward | CATGTACGTTGCTATCCAGGC |
|  | Reverse | CTCCTTAATGTCACGCACGAT |

**Supplementary Figures**

**Supplementary Figure 1**


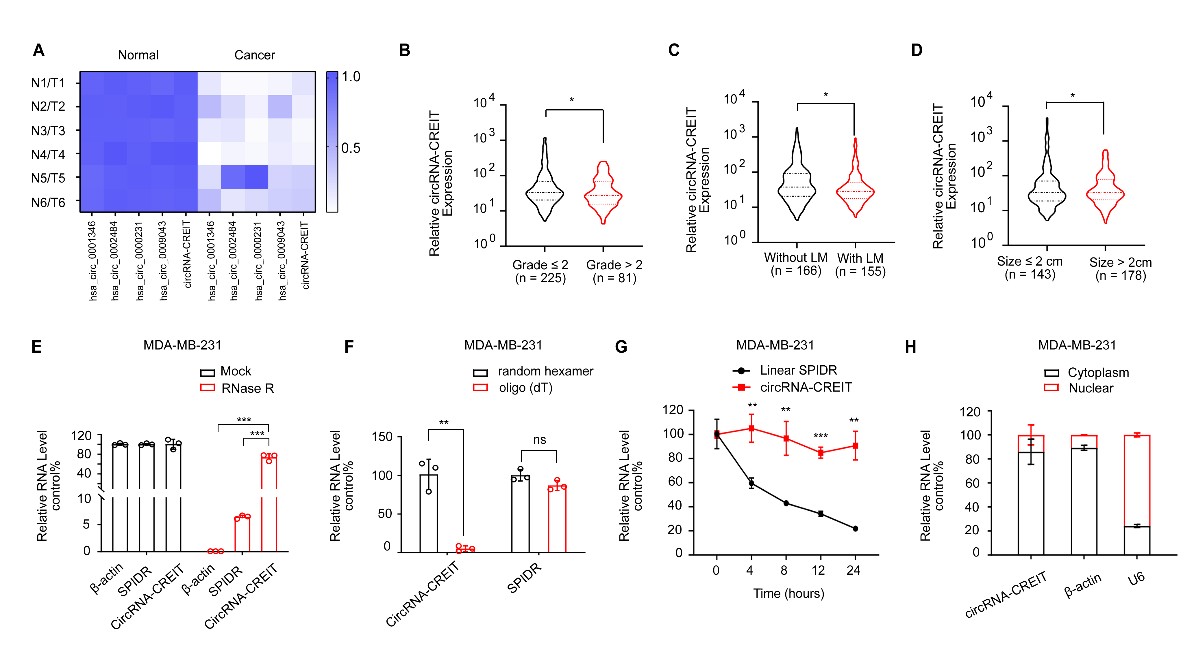


**Supplementary Figure 1.** (A) The heatmap showed the expression of five candidate circRNAs in breast cancer tissues and paired normal counterparts. (B) Comparison of expression levels of circRNA-CREIT between breast cancer tissues with pathological grade > 2 and grade ≤ 2. (C) Comparison of expression levels of circRNA-CREIT between breast cancer tissues with and without lymph node metastasis. (D) Comparison of expression levels of circRNA-CREIT between breast cancer tissues with tumor size > 2 cm and tumor size ≤ 2 cm. (E) qRT-PCR analysis of the expression of circRNA-CREIT, linear SPIDR and β-actin in TNBC cells with or without RNase R treatment. (F) qRT-PCR analysis of circRNA-CREIT expression in the cDNA reversely transcribed with random hexamer or oligo (dT) primers. The expression levels of circRNA-CREIT using random hexamer primers were as control. (G) Relative RNA levels of circRNA-CREIT and linear SPIDR after Actinomycin D treatment detected by qRT-PCR. (H) Detection of circRNA-CREIT expression in cytoplasmic and nuclear fractions of RNAs extracted from TNBC cells. ns, no significance; *, *p* < 0.05; **, *p* < 0.01; ***, *p* < 0.001 compared with the controls.


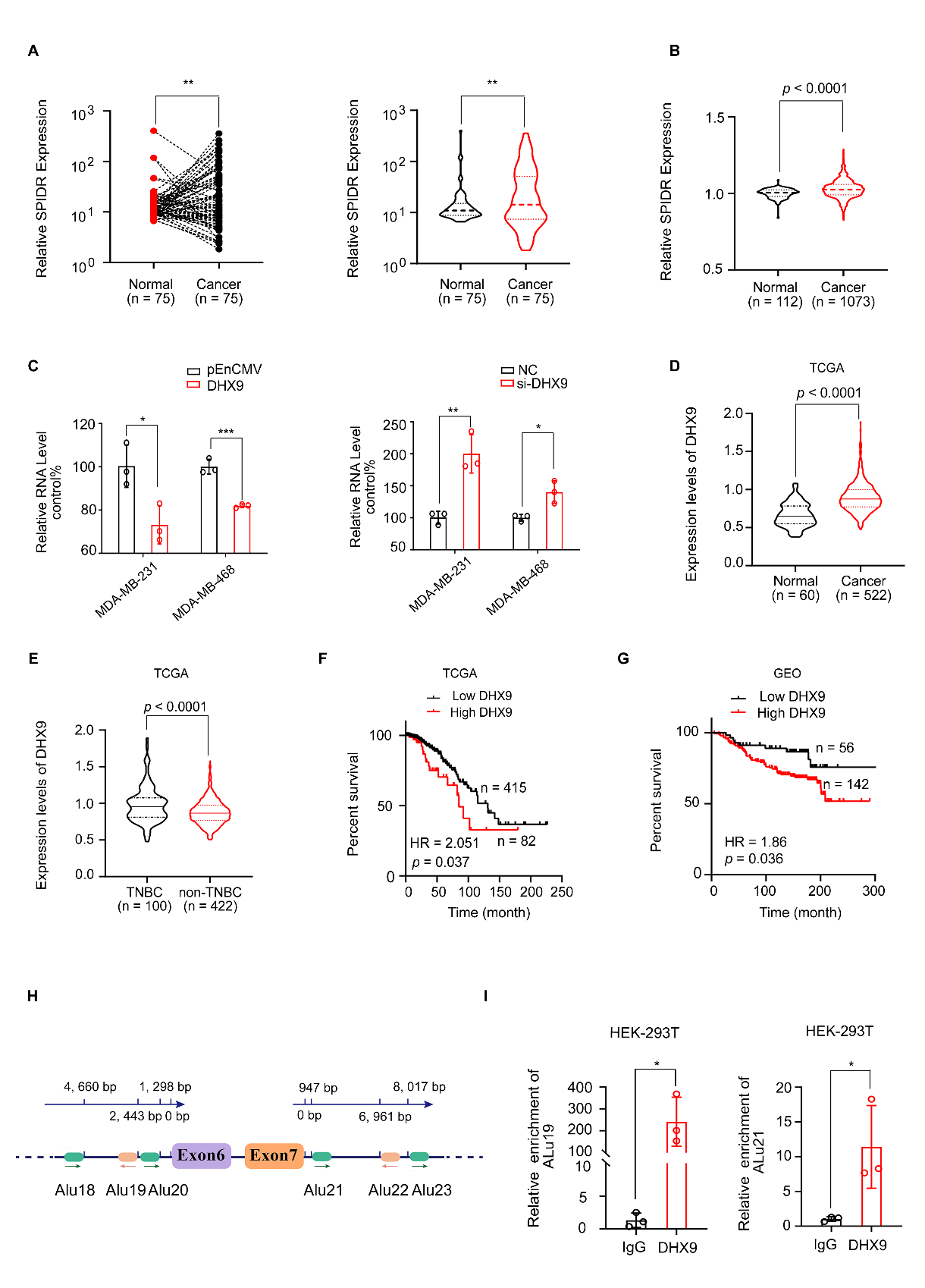
**Supplementary Figure 2**

**Supplementary Figure 2. The biogenesis of circRNA-CREIT was inhibited by DHX9.** （A）Expression of SPIDR mRNA in 75 breast cancer tissues and paired normal mammary tissues detected by qRT-PCR assay. (B) Violin plot showing SPIDR mRNA expression in breast cancer tissues and normal mammary tissues from the TCGA database. (C) The expression levels of circRNA-CREIT after DHX9 overexpression or silencing were detected by qRT-PCR assays. (D) Violin plot showing DHX9 expression in breast cancer tissues and normal mammary tissues from the TCGA database. (E) The violin plot showed the expression level of DHX9 in TNBC and non-TNBC subtypes from TCGA database. (F) Kaplan–Meier survival analysis of DHX9^high^ and DHX9^low^ breast cancer patients from TCGA database. (G) Kaplan–Meier survival analysis of DHX9^high^ and DHX9^low^ breast cancer patients from GEO database (GSE7390). (H) The schematic diagram indicating the distribution (lower) of Alu elements located at the flanking introns of circRNA-CREIT and the distances to the back-splicing junction site (upper). (I) The interaction between DHX9 and the Alu elements was validated by RIP assays. Three independent experiments were conducted for each result. ns, no significance; *, *p* < 0.05; **, *p* < 0.01; ***, *p* < 0.001 compared with the controls.

**Supplementary Figure 3**


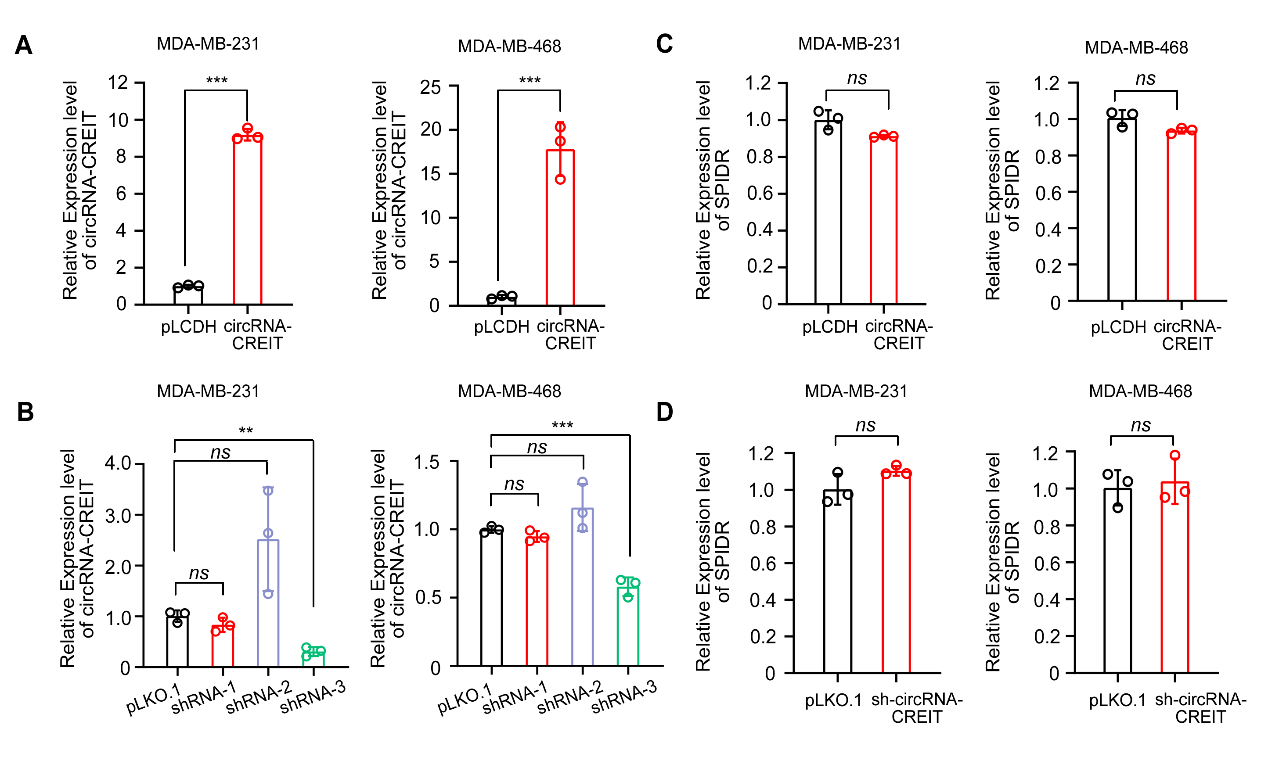


**Supplementary Figure 3.** **The overexpression and knockdown efficiencies of circRNA-CREIT.** (A, B) The overexpression and knockdown efficiencies of circRNA-CREIT in TNBC cells were detected by qRT-PCR assays. (C, D) qRT-PCR assays showed circRNA-CREIT had no effects on the expression of its host gene. Three independent experiments were conducted for each result. ns, no significance; **, *p* < 0.01; ***, *p* < 0.001 compared with the controls.

**Supplementary Figure 4**


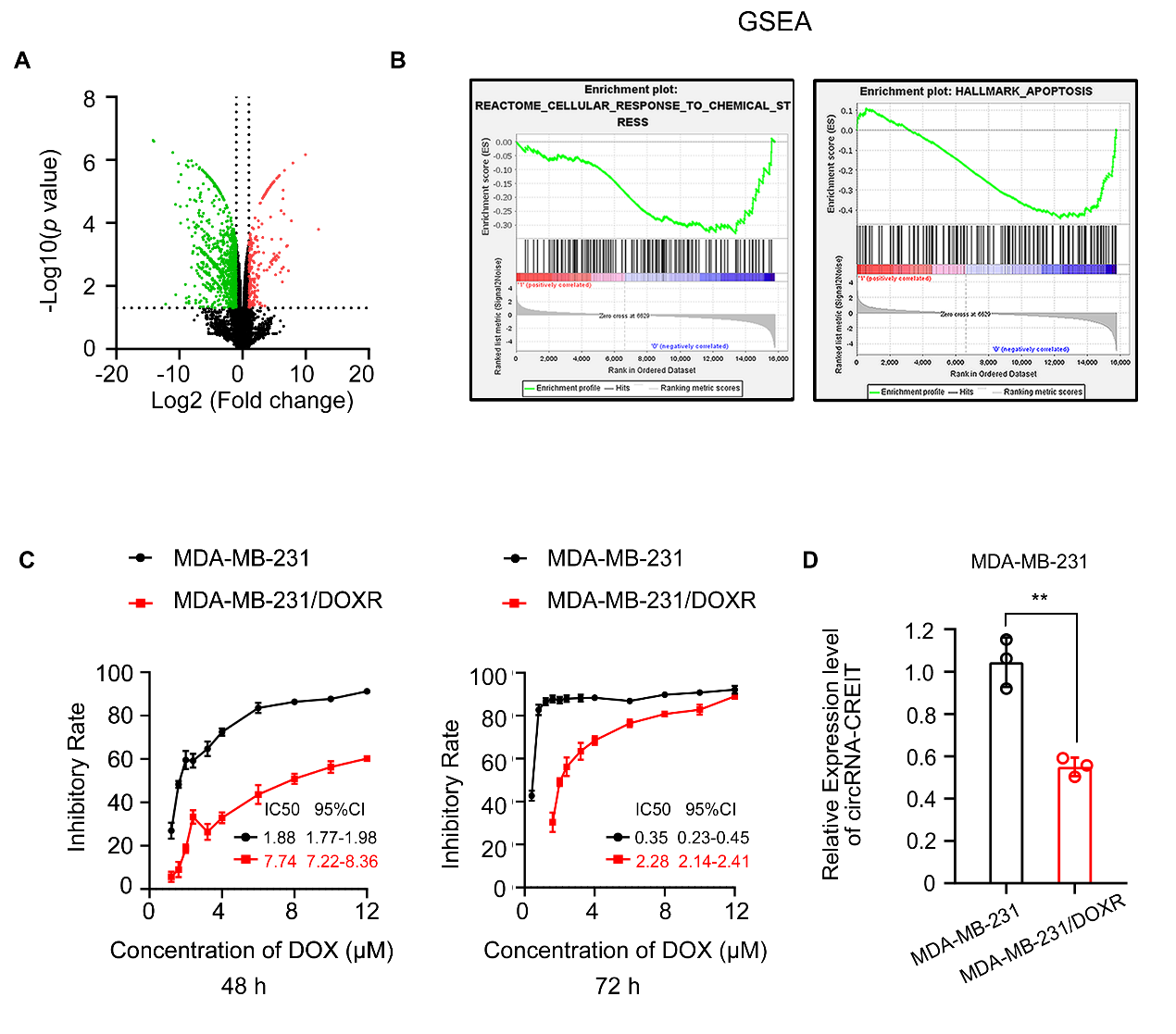


**Supplementary Figure 4.** **circRNA-CREIT** **was correlated with stress response and chemoresistance in TNBC.** (A) Differentially expressed genes in circRNA-CREIT overexpressed MDA-MB-231 cells were screened based on the RNA-seq data. (B) GSEA analysis indicated circRNA-CREIT might play a role in regulation of cellular response to chemical stress and cell apoptosis. (C) MTT assay was performed to validate the resistance to DOX of MDA-MB-231/DOXR cells. The parental MDA-MB-231 cell were used as control. (D) The expressions of circRNA-CREIT in MDA-MB-231 and MDA-MB-231/DOXR cells were detected by qRT-PCR assays. Three independent experiments were conducted for each result. **, *p* < 0.01 compared with the controls.

**Supplementary Figure 5**


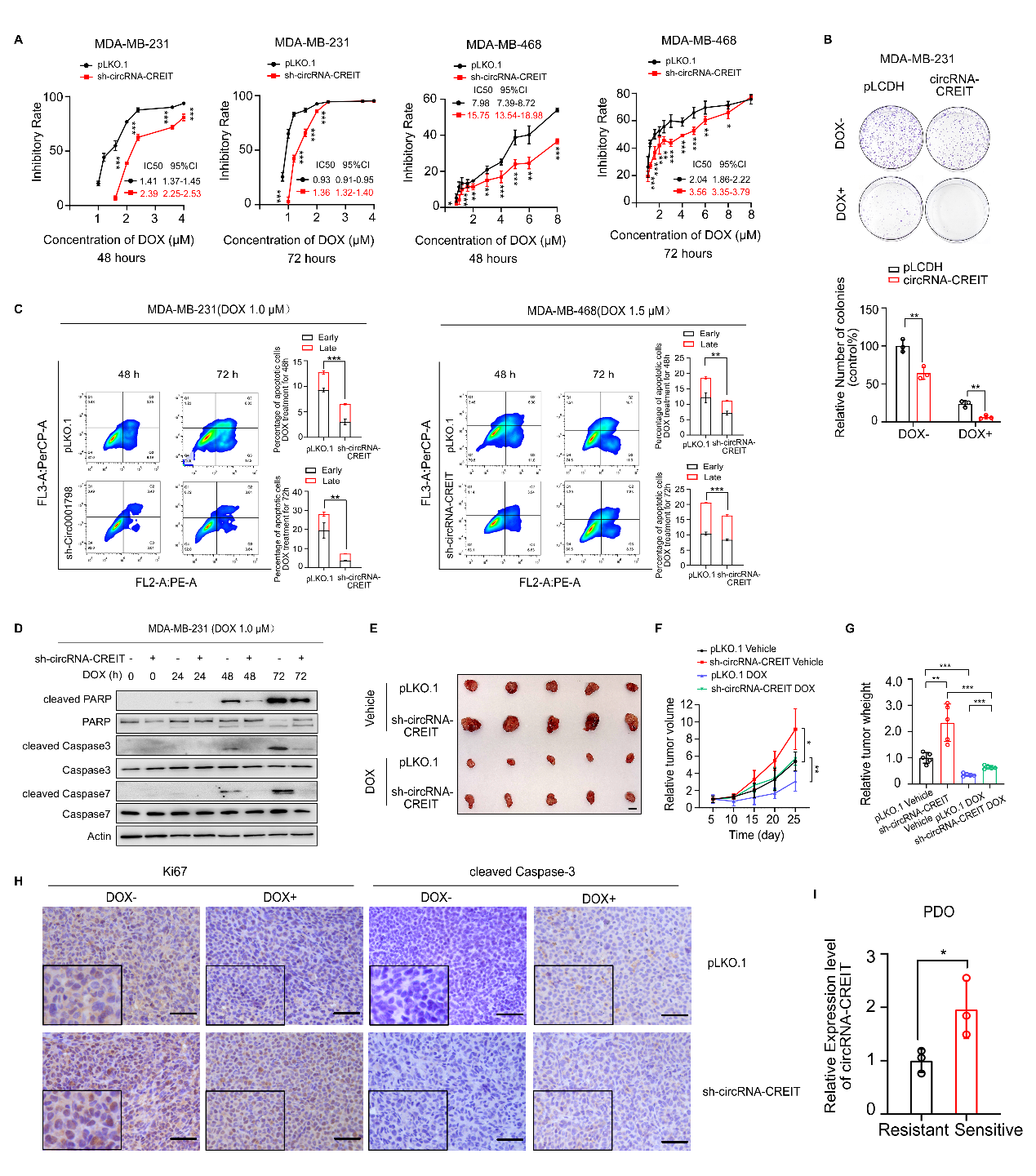


**Supplementary Figure 5. circRNA-CREIT significantly enhanced chemosensitivity of TNBC *in vitro* and *in vivo*.** (A) The impacts of circRNA-CREIT knockdown on the cytotoxic effects of DOX was shown. Cells were exposed to increasing concentrations of DOX and cell viability was detected by MTT assay. IC50 value in company with the 95% CI was presented. (B) The effect of circRNA-CREIT on enhancing TNBC chemosensitivity was detected by colony formation assay. (C) Suppressed cell apoptosis by circRNA-CREIT knockdown in MDA-MB-231 and MDA-MB-468 cells was demonstrated via flow cytometry. (D) Western blotting analysis for expression of apoptosis pathway markers in circRNA-CREIT knockdown and control TNBC cells under DOX treatment. (E) Photographs showing the xenograft tumors subcutaneously injected with TNBC cells transfected with sh-circRNA-CREIT or empty vectors. Scale bars = 10 mm. (F-G) Growth curves and tumor weights of xenograft tumors in the sh-circRNA-CREIT group and empty vector group. (H) Representative images of IHC staining for Ki67 and cleaved caspase-3 in different groups. Scale bars = 100 μm. (I) The columns showing the relative expression levels of circRNA-CREIT in the chemoresistant-PDO (PDO 3, 4 and 6) and chemosensitive-PDO (PDO, 1, 2 and 5) groups. Three independent experiments were conducted for each result. *, *p* < 0.05; **, *p* < 0.01; ***, *p* < 0.001 compared with the controls.


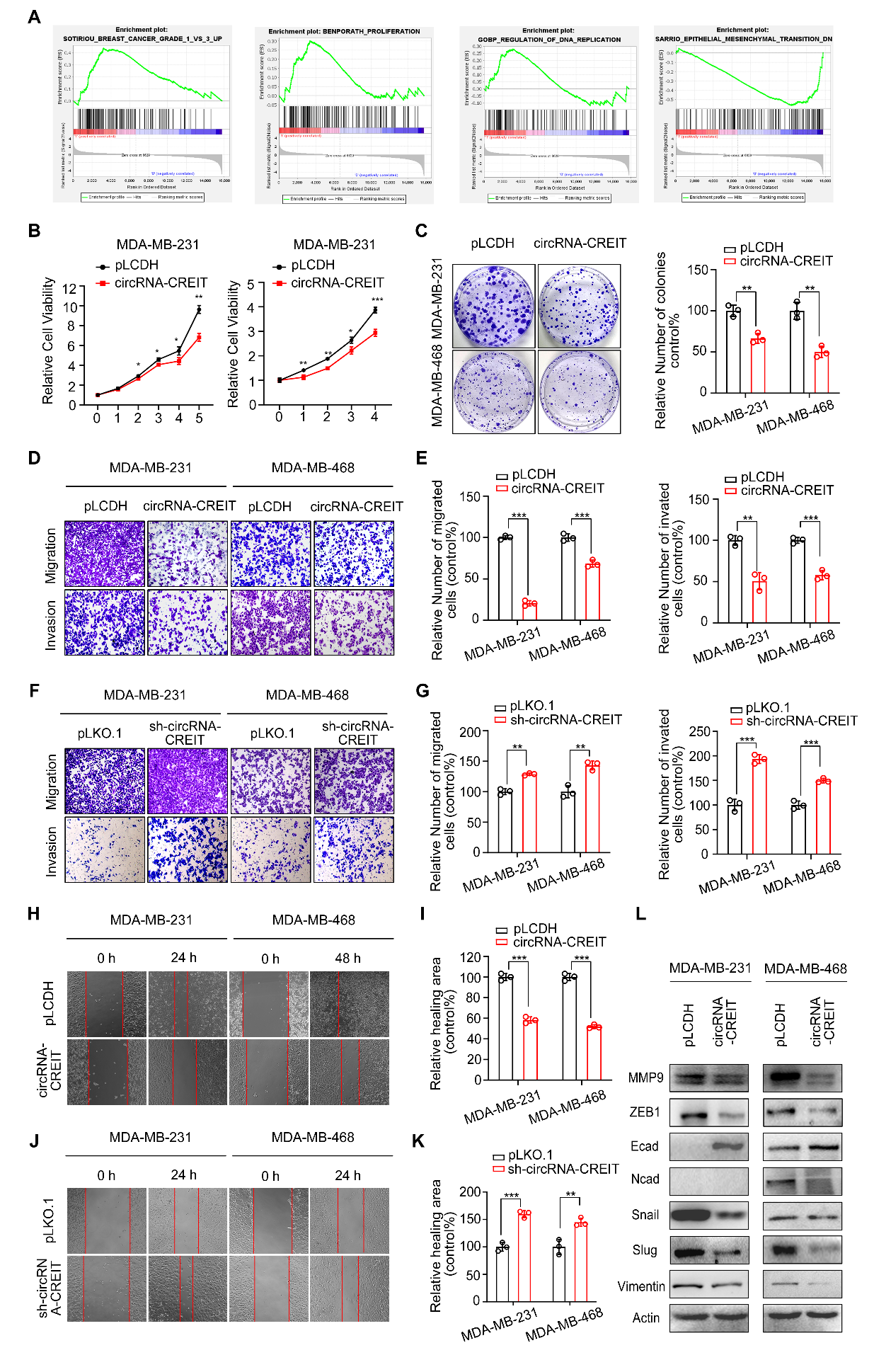
**Supplementary Figure 6**

**Supplementary Figure 6. circRNA-CREIT significantly inhibited cell growth, migration and invasion of TNBC cells.** (A) GSEA analysis indicated circRNA-CREIT might play a role in breast cancer progression, cell proliferation, DNA replication and EMT process. (B, C) MTT assay and colony formation assay showed circRNA-CREIT significantly inhibited proliferation in TNBC cells. (D-G) Transwell assay showed the inhibitory roles of circRNA-CREIT in suppressing TNBC cell migration and invasion. (H-K) Wound healing assay demonstrated circRNA-CREIT overexpression significantly inhibited TNBC cell migration, contrary to the effects of circRNA-CREIT knockdown. (L) Western blotting analysis for the effect of circRNA-CREIT overexpression on the expression of EMT markers in TNBC cells. Three independent experiments were conducted for each result. *, *p* < 0.05; **, *p* < 0.01; ***, *p* < 0.001 compared with the controls.

**Supplementary Figure 7**


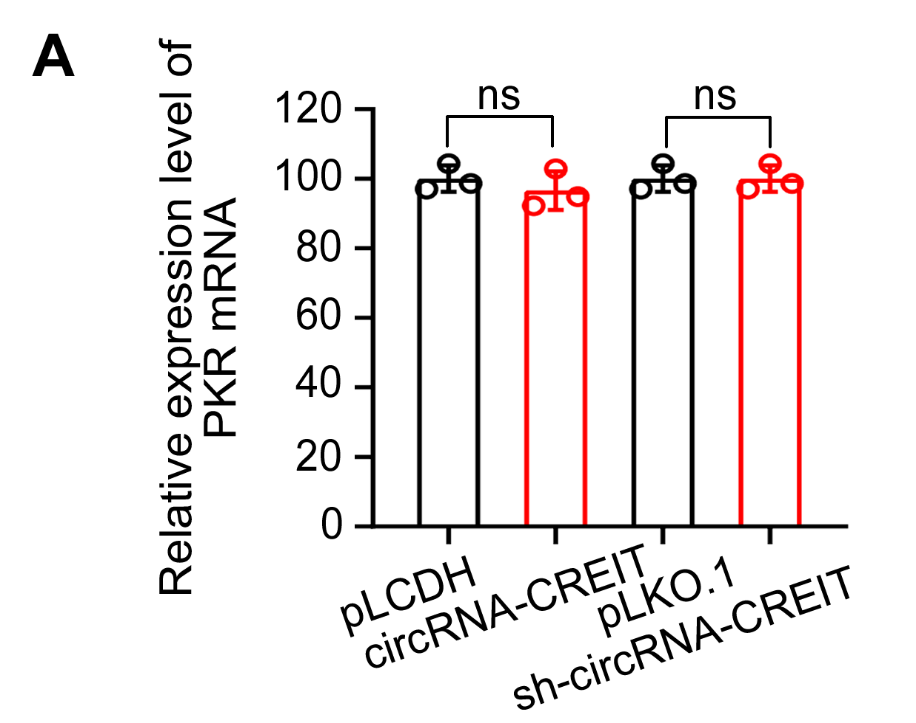


**Supplementary Figure 7.** **The expression levels of PKR mRNAs were not affected by circRNA-CREIT.** (A) qRT-PCR assay showed circRNA-CREIT had no effect on the expression of PKR at mRNA level in MDA-MB-231 cells. Three independent experiments were conducted for each result. ns, no significance compared with the controls.

**Supplementary Figure 8**


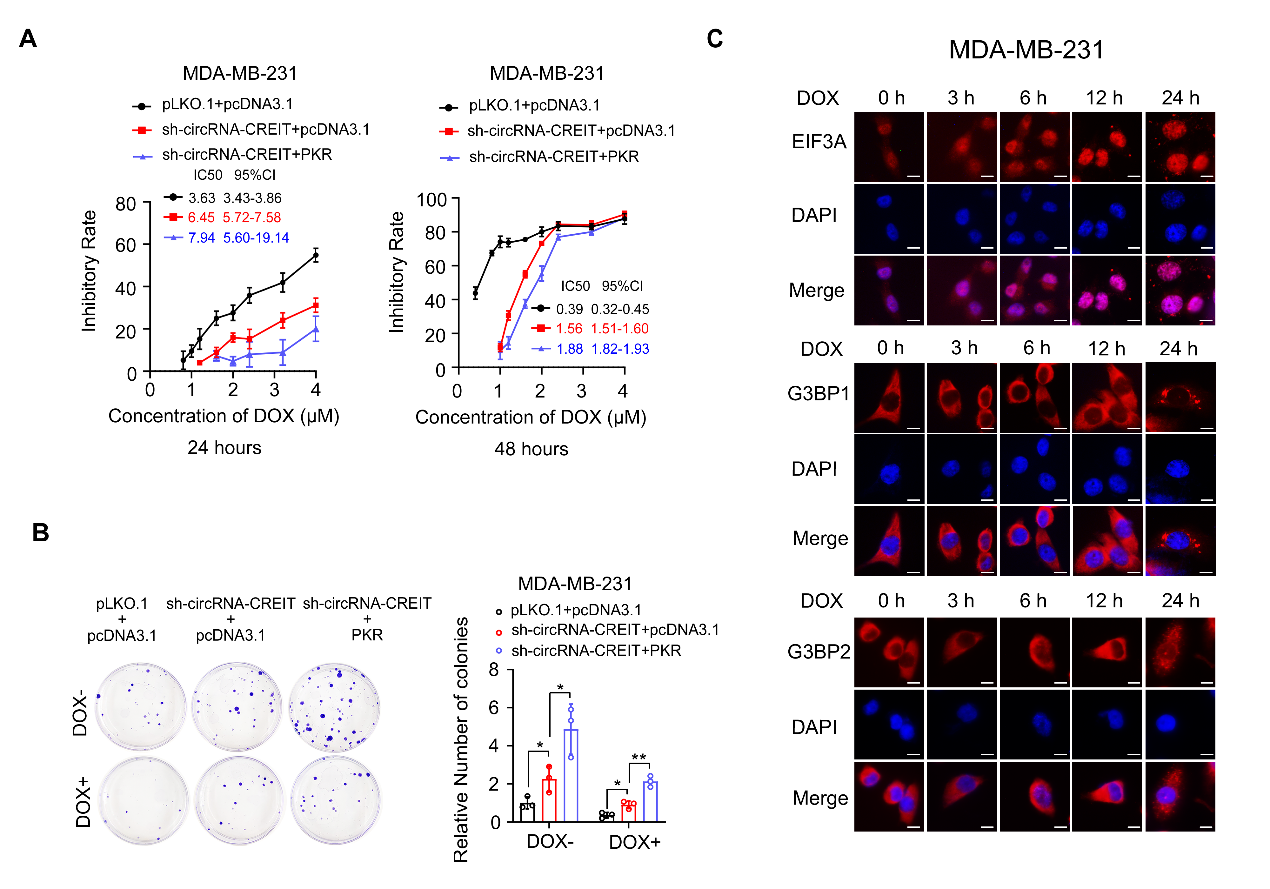


**Supplementary Figure 8.** (A) MTT assay showing the synergistic effects of PKR overexpression and circRNA-CREIT knockdown on enhancing cell chemoresistance. IC50 and the 95% CI of cells with different treatment were shown. (B) Colony formation assays presenting the synergistic roles of PKR and circRNA-CREIT knockdown on enhancing cell chemoresistance. (C) The immunofluorescence assay showed the formation of stress granules induced by DOX treatment for indicated time. EIF3A, G3BP1 and G3BP2 were served as classical markers of SGs. Three independent experiments were conducted for the result. Scale bars = 10 μm *, *p* < 0.05; **, *p* < 0.01 compared with the controls.

**Supplementary Figure 9**


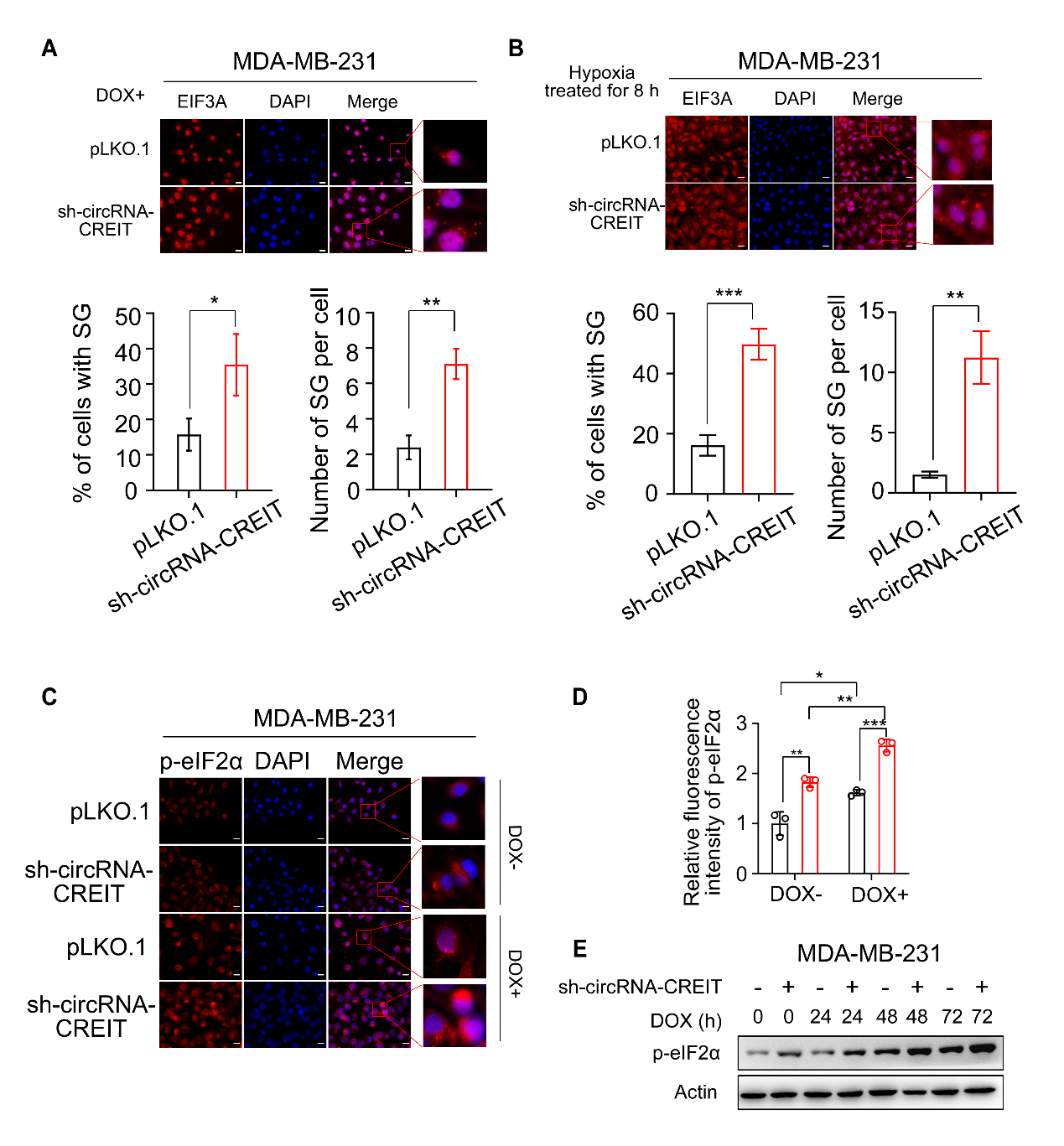


**Supplementary Figure 9.** **circRNA-CREIT knockdown enhanced SGs formation by promoting the phosphorylation of eIF2α.** (A, B) The immunofluorescence assay showed circRNA-CREIT knockdown could enhance the formation of DOX-induced and hypoxia-induced SGs. Scale bars = 20 μm. EIF3A was used as the SGs marker. Scale bars = 20 μm. EIF3A was used as the SGs marker. Three independent experiments were conducted for each result. (C, D) Immunofluorescence staining for p-eIF2α after circRNA-CREIT knockdown with or without DOX treatment (for 24 h). Quantitative analyses were performed using Image J. (E) Western blotting assay showed the effects of sh-circRNA-CREIT and DOX treatment on p-eIF2α expression levels. Three independent experiments were conducted for the result. *, *p* < 0.05; **, *p* < 0.01; ***, *p* < 0.001 compared with the controls.

**Supplementary Figure 10**


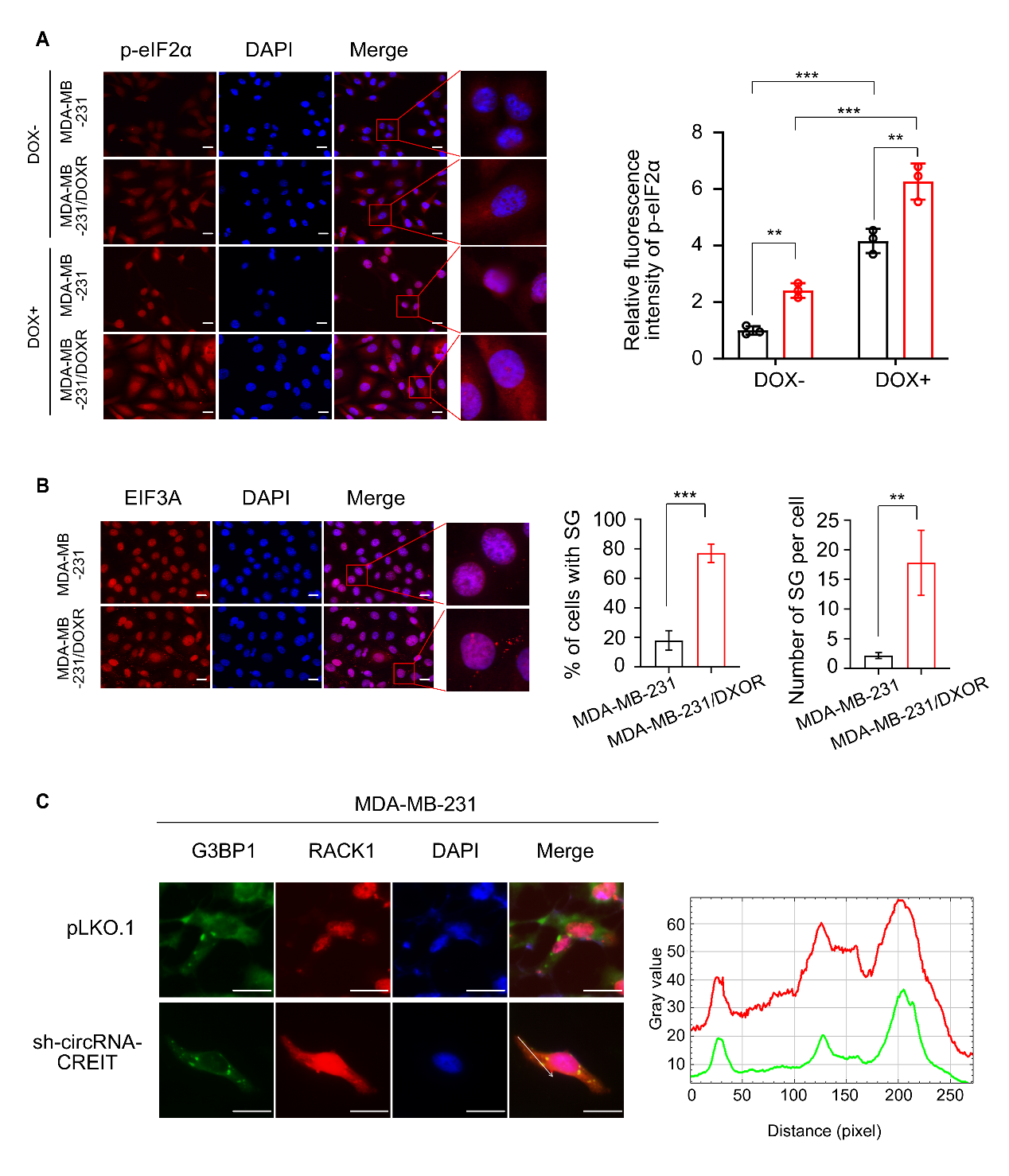


**Supplementary Figure 10.** (A) The immunofluorescence assay showed the higher expression level of p-eIF2α in MDA-MB-231/DOXR cells with or without DOX treatment. Scale bars = 20 μm. (B) The immunofluorescence assay showed more stress granules were induced in MDA-MB-231/DOXR cells under DOX (1.0 μM) treatment for 24 h. Scale bars = 20 μm. (C) Subcellular localization of RACK1 (red) and the SGs marker G3BP1 (green) under DOX treatment. Cells were transfected with RACK1-pmCherry-C1 plasmids, then treated with DOX for 24 h and subjected to immunofluorescence. Scale bars = 20 μm. Three independent experiments were conducted for the result. **, *p* < 0.01; ***, *p* < 0.001 compared with the controls.


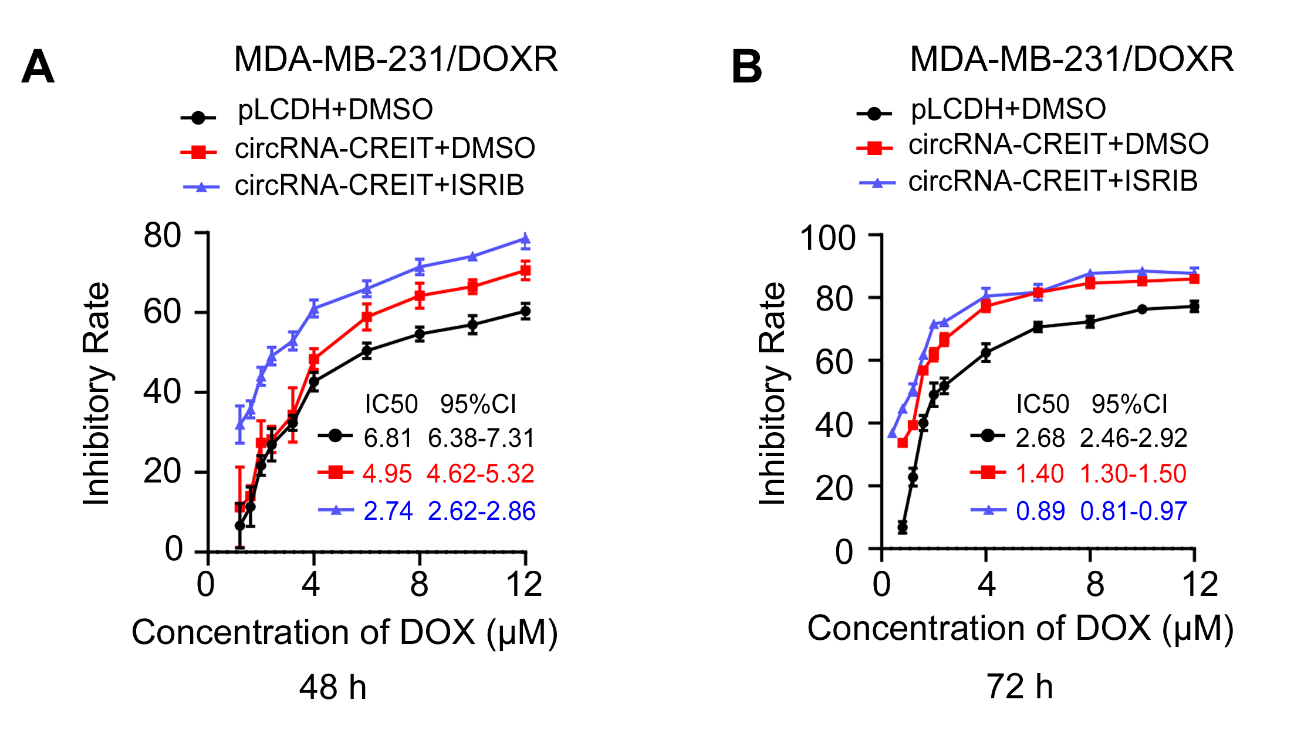
**Supplementary Figure 11**

**Supplementary Figure 11.** (A, B) MTT assay showed the synergistic roles of circRNA-CREIT and ISRIB in reversing chemoresistance in DOX-resistant TNBC cells. The cell viability was detected with MTT assays. IC50 and the 95% CI of cells with different treatment were shown. Three independent experiments were conducted for each result.

**Supplementary Figure 12**


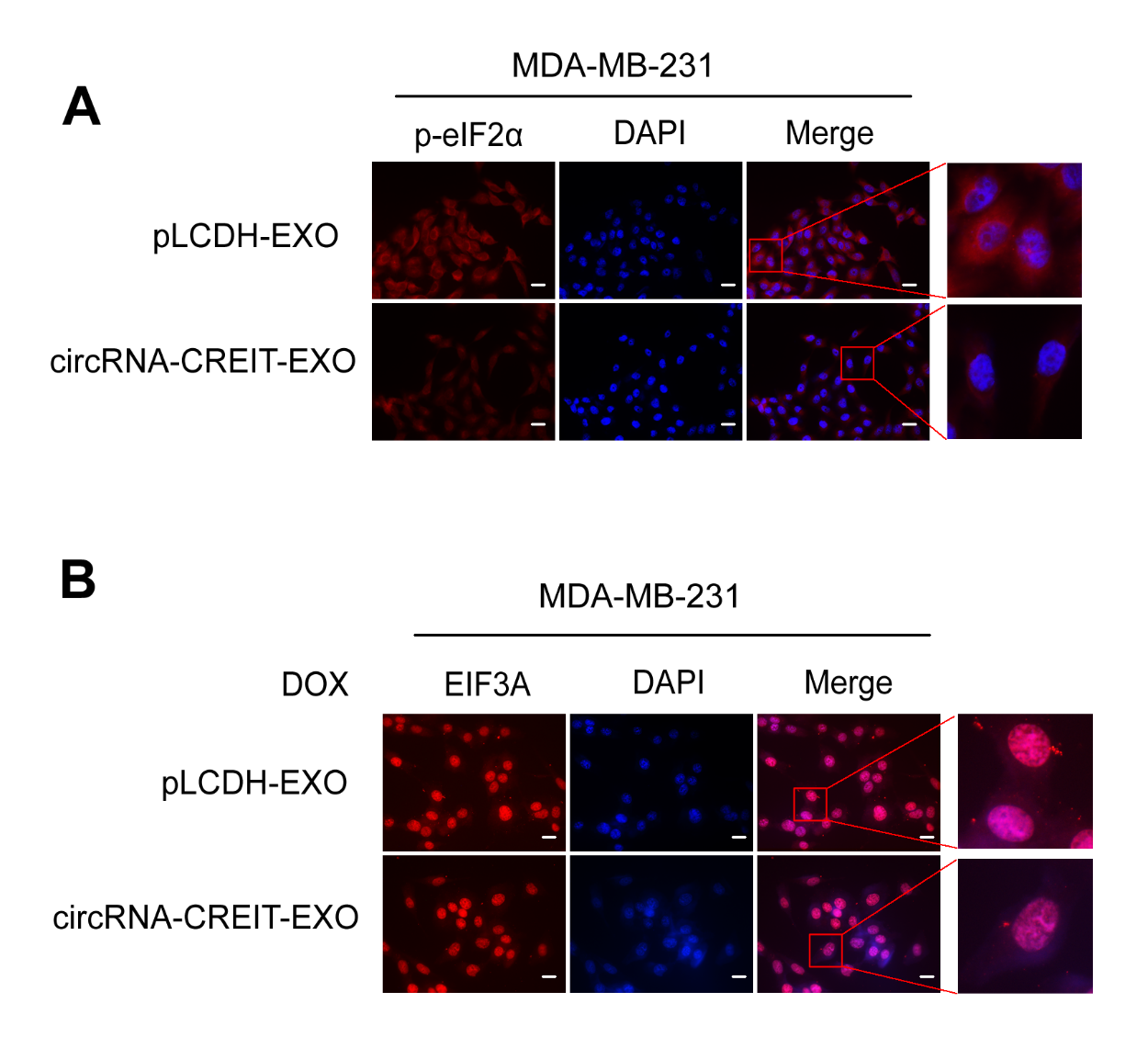


**Supplementary Figure 12.** (A) Treatment with circRNA-CREIT-EXO led to the downregulation of p-eIF2α in MDA-MB-231 cells. Scale bars = 20 μm. (B) circRNA-CREIT-EXO inhibited the formation of SGs induced by DOX (1.0 μM) treatment for 24 h. Scale bars = 20 μm. Three independent experiments were conducted for each result.
